# Unveiling the photocatalytic potential of graphitic carbon nitride (g-C_3_N_4_): a state-of-the-art review

**DOI:** 10.1039/d4ra04234d

**Published:** 2024-08-15

**Authors:** Mahmoud A. Ahmed, Safwat A. Mahmoud, Ashraf A. Mohamed

**Affiliations:** a Chemistry Department, Faculty of Science, Ain Shams University Cairo-11566 Egypt mahmoudmahmoud_p@sci.asu.edu.eg; b Physics Department, Faculty of Science, Northern Border University Arar 13211 Saudi Arabia

## Abstract

Graphitic carbon nitride (g-C_3_N_4_)-based materials have emerged as promising photocatalysts due to their unique band structure, excellent stability, and environmental friendliness. This review provides a comprehensive and in-depth analysis of the current state of research on g-C_3_N_4_-based photocatalysts. The review summarizes several strategies to improve the photocatalytic performance of pristine g-C_3_N_4_, *e.g.*, by creating heterojunctions, doping with non-metallic and metallic materials, co-catalyst loading, tuning catalyst morphology, metal deposition, and nitrogen-defect engineering. The review also highlights the various characterization techniques employed to elucidate the structural and physicochemical features of g-C_3_N_4_-based catalysts, as well as their applications of in photocatalytic degradation and hydrogen production, emphasizing their remarkable performance in pollutants' removal and clean energy generation. Furthermore, this review article investigates the effect of operational parameters on the catalytic activity and efficiency of g-C_3_N_4_-based catalysts, shedding light on the key factors that influence their performance. The review also provides insights into the photocatalytic pathways and reaction mechanisms involving g-C_3_N_4_ based photocatalysts. The review also identifies the research gaps and challenges in the field and presents prospects for the development and utilization of g-C_3_N_4_-based photocatalysts. Overall, this comprehensive review provides valuable insights into the synthesis, characterization, applications, and prospects of g-C_3_N_4_-based photocatalysts, offering guidance for future research and technological advancements in this rapidly growing field.

## Abstract 2

Graphitic carbon nitride (g-C_3_N_4_)-based materials have emerged as promising photocatalysts due to their unique band structure, excellent stability, and environmental friendliness.

## Graphical abstract

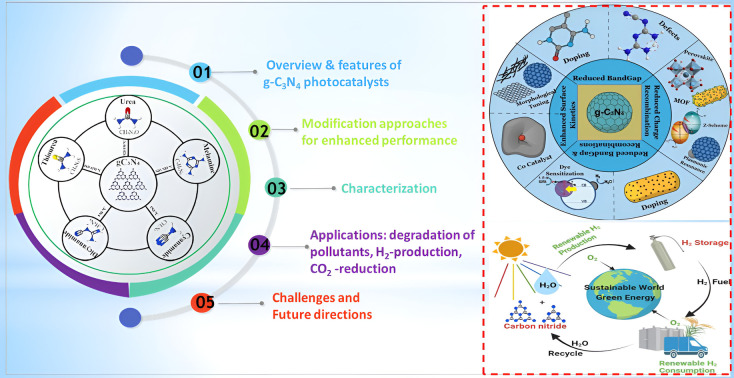

## 1 Introduction

The discharge of pollutants into aquatic environments has increased significantly as a result of the manufacturing sector's growth.^1,2^ Because of their hazardous characteristics and possible carcinogenicity, organic pollutants found in both air and water are especially concerning.^3^ The chemical processing industries, building materials, textile production, and coatings used in indoor furniture are the main producers of these pollutants.^4,5^ Exposure to organic pollutants, whether indoors or outdoors, has been associated with various adverse health effects including hypertension, renal damage, Alzheimer's disease, nausea, epilepsy, mental confusion, and vomiting.^4–6^ Furthermore, the mutagenic and carcinogenic impacts of these pollutants are noteworthy.^7,8^ Moreover, organic pollutants, such as dyes, pesticides, pharmaceuticals, phenols, and others, significantly impact the receiving water bodies by changing key variables like unpleasant odors, color, toxicity levels, biochemical oxygen demand (BOD), and chemical oxygen demand (COD). Some of these organic pollutants have long half-life times, (bio)accumulate, are not easily degraded, and damage the marine flora and fauna, aquatic lives, and ultimately human health. In addition to these environmental concerns, the global community also faces a pressing challenge in terms of ensuring energy security.^9^ Fossil fuels are limited resources, and using them to produce energy increases harm to the atmosphere by emitting various pollutants, including carbon dioxide.^10^ This has spurred a global effort to explore technologies that promote the utilization of renewable energy sources and address environmental challenges.^11^

On the other hand, water purification has been achieved using conventional techniques such as reverse osmosis, adsorption, membrane filtration, precipitation, coagulation, ion exchange, and biological treatments.^12–17^ However, when handling complicated pollutants with a variety of chemical and physical features, these conventional approaches have limits in terms of efficiency and energy usage, as well as the increased risk of generating secondary pollutants.^18^ Nevertheless, in advanced oxidation processes (AOP), photocatalysis approach has emerged as a cost-effective, trustworthy, and environmentally benign alternative.^19–22^ This approach utilizes solar radiation to facilitate various applications, including treating pollutants, facilitating chemical reactions, and splitting water to produce hydrogen.^23–27^ The efficient utilization of solar photocatalysis holds significant research value in terms of improving the environment and reducing greenhouse gas emissions. Typically, a photocatalytic process involves stages, such as harnessing visible light, exciting photocarriers, segregating and migrating photo-induced charge carriers to active sites, and facilitating the redox process on the photocatalyst surface.^28,29^ These redox processes are responsible for generating reactive species, such as superoxide radicals (˙O_2_^−^), and hydroxyl radicals (˙OH) which play a key role in the overall photocatalytic process^30,31^

Recently, two-dimensional (2D) compounds like graphitic carbon nitride (g-C_3_N_4_), graphene, boron nitride, and transition-metal dichalcogenides, with excellent features have been widely employed in chemical sensors, electronic and optical devices, energy storage and generation, as well as environmental remediation.^32–34^ In particular, g-C_3_N_4_, a metal-free polymer semiconductor containing tri-*s*-triazine units, has garnered a great deal of interest due to its potential uses in photochemistry and photocatalysis.^35^

Graphitic carbon nitride (g-C_3_N_4_) is regarded as one of the first organic conjugated polymers, having been discovered in 1834.^36^ There are five primary phases that g-C_3_N_4_ may be categorized into: the cubic phase, the pseudo-cubic phase, the graphitic phase with minimal compressibility and remarkable hardness that is comparable to a diamond, the α-phase, and the β-phase.^37^ Research communities have become quite excited by g-C_3_N_4_-based materials as photocatalysts because of its non-toxicity, high visible light harvesting, π-conjugated assembly, increased profusion, and chemical and thermal durability.^38,39^ The optical bandgap of g-C_3_N_4_ at 2.7 eV (460 nm), with VB and CB potentials at −1.09 and +1.56 V (*vs.* NHE), respectively, make g-C_3_N_4_ attractive material for overall water splitting.^40,41^ Furthermore, the widespread usage of g-C_3_N_4_-based materials as a visible-light-driven photocatalyst is mostly due to its easy synthesis process from readily accessible, affordable precursors.^42,43^ Additionally, g-C_3_N_4_ has a powerful electrical conductivity and distinct conjugated structure due to the graphitic stacking of g-C_3_N_4_ layers connected by tertiary amines.^44,45^ The presence of carbon and nitrogen atoms with distinct valence states results in the creation of multiple band structures; therefore, pristine g-C_3_N_4_ has shown promise as a photocatalyst, but it also has limitations that must be addressed.^43,46^ One major limitation is its low photocatalytic activity, attributed to its wide bandgap energy, which limits its absorption of the solar spectrum.^47^ Additionally, the performance of photocatalytic techniques is further decreased by the quick coupling of photo-generated charge carriers in g-C_3_N_4_.^48^ It also has limited charge carrier mobility, hindering efficient charge transfer. Other limitations of pristine g-C_3_N_4_ are its relatively low specific surface area and lack of stability under photocatalytic conditions, as prolonged exposure to light and reactive species can degrade its performance over time. To overcome these limitations, different modification approaches were adopted to enhance the performance of pure g-C_3_N_4_ including heterojunctions, doping, co-catalyst loading, tuning morphology, metal deposition, and defect engineering.^49–52^

Heterostructure development has emerged as the most promising approach to improve the photocatalytic activity of g-C_3_N_4_. One of the advantageous properties of g-C_3_N_4_ is its tunable band gap, which allows precise control over the energy levels of its highest occupied molecular orbital (HOMO) and lowest unoccupied molecular orbital (LUMO).^53^ This tunability significantly impacts the photoelectronic performance of g-C_3_N_4_ as a photocatalytic nanosheet. By constructing hetero structures, the band gap of g-C_3_N_4_ can be effectively modified, leading to expanded light harvesting and promoting the separation of hole–electron pairs.^54,55^ This modification approach involves the intentional introduction of metal, nonmetal, or other nanomaterials into the structure, offering a means to enhance the photocatalytic performance of g-C_3_N_4_.

**Fig. 1:**
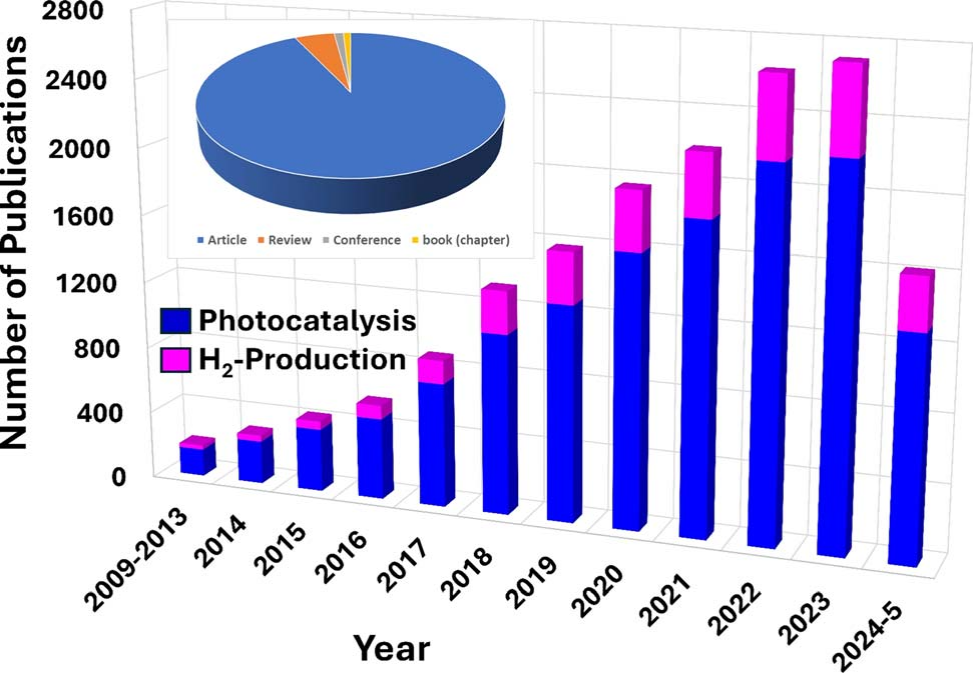
Number of publications in Scopus database reporting g-C_3_N_4_-based composites for photocatalytic and H_2_-production applications: keywords “(g-C_3_N_4_) and ((photocataly*) or (hydrogen production))”.

Thus, recent research has highlighted the potential of g-C_3_N_4_ composites in effectively removing various pollutants from wastewater, such as dyes, oil spills, heavy metal ions, pesticides, microplastics, phenols, and pharmaceuticals.^54,56–59^ Moreover, there is increasing research interest in utilizing g-C_3_N_4_-composites for hydrogen generation.^60^ The number of publications focusing on pollutant removal and H_2_-production using g-C_3_N_4_ nanocomposites has shown a notable increase over the last few years, as seen in Fig. 1. Initially, there were only a few publications per year, indicating limited attention to the topic. However, since 2017, there has been a rapid upward trend in both citations and publications, signifying a growing interest in the field, where documents on photocatalysis were almost five times higher than those on H_2_-production. Most of these publications consist of journal articles (93%), with a smaller fraction being reviews (4.9%), and conference articles (1.1%), as shown in Fig. 1. This indicates a scarcity of dedicated and updated review papers, which are essential for providing interested researchers and the scientific community with a comprehensive and up-to-date evaluation of g-C_3_N_4_-composites' application as photocatalysts.

This comprehensive review aims at providing a detailed examination of the synthesis methods of g-C_3_N_4_-based photocatalysts, along with their applications in environmental remediation, *e.g.*, organic pollutants' degradation and hydrogen production. Additionally, the review highlights the characterization techniques used to understand the crystal structure, morphology, surface area, nanoparticle distribution, and compositional properties of g-C_3_N_4_-based photocatalysts. Moreover, the review describes the mechanisms and factors influencing the photocatalytic performance of g-C_3_N_4_-based photocatalysts in organic pollutant degradation, providing insights into the identification of key intermediates and reactive species involved in the photocatalytic degradation processes. It further investigates the strategies employed to enhance the efficiency and selectivity of g-C_3_N_4_-based photocatalysts, including the utilization of metal cocatalysts, co-doping techniques, heterojunction formation, and surface modification. Additionally, the review assesses the g-C_3_N_4_-based photocatalysts' application in hydrogen production through water splitting, evaluating their performance in terms of hydrogen evolution rate, stability, and selectivity, while discussing the underlying mechanisms of photogenerated charge separation and transfer.

## 2 Modification of g-C_3_N_4_ for improved photocatalytic activity

Composite g-C_3_N_4_ photocatalysts have gained significant attention in recent years due to their potential for efficient and sustainable energy conversion and environmental remediation. The g-C_3_N_4_ modification with other materials allows for improved light absorption, better charge separation, and boosted catalytic performance, resulting in enhanced photocatalytic activity.

**Scheme 1:**
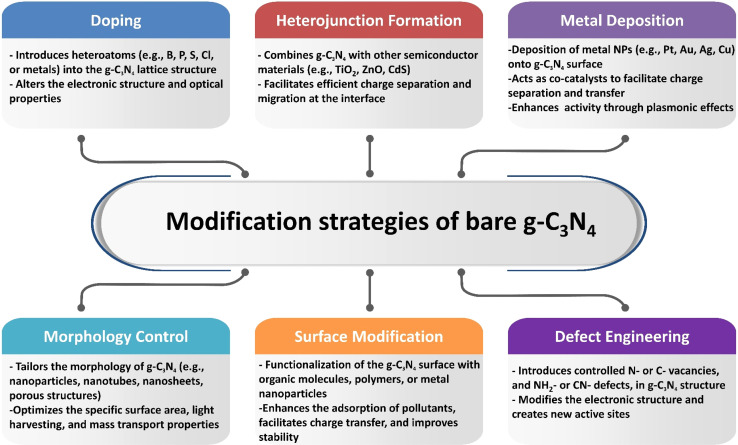
Modification methods of g-C_3_N_4_ to enhance its photocatalytic performance.

Several approaches have been applied to modify pristine graphitic carbon nitride and improve its photocatalytic performance, such as creating heterojunctions, doping with non-metallic and metallic materials, co-catalyst loading, tuning catalyst morphology, metal deposition, and nitrogen-defect engineering, as shown in Scheme 1.^49–52,61,62^ When it comes to the fabrication of g-C_3_N_4_ composites as photocatalysts, two main approaches are commonly employed based on the crystallization process: *in situ* crystallization and *ex situ* crystallization.

### 2.1. Synthesis of g-C_3_N_4_ composites by *in situ* crystallization

*In situ* crystallization: the g-C_3_N_4_ composite is fabricated by incorporating the other material during the polymerization process of g-C_3_N_4_ itself. This approach involves the co-condensation of a precursor monomer of g-C_3_N_4_ with other components, which subsequently polymerizes and crystallizes simultaneously.^63,64^ During the *in situ* crystallization process, the precursor monomers of g-C_3_N_4_, typically urea, thiourea, melamine, cyanamide, or dicyanamide are combined with the desired components, such as metal precursors or carbon-based materials. The mixture is then subjected to thermal treatment under specific temperature and atmosphere conditions. The heating process triggers the polymerization and condensation of the monomers into a layered g-C_3_N_4_ structure, thereby incorporating the additional components into the composite. *In situ* crystallization offers several advantages, including uniform distribution of the composite components and good interfacial interaction between g-C_3_N_4_ and the additional material. This approach allows for control over the composition and structure of the composite, leading to improved photocatalytic performance.^65,66^

### 2.2. Synthesis of g-C_3_N_4_ composites by *ex situ* crystallization

In *ex situ* Crystallization, g-C_3_N_4_ is synthesized separately, and subsequently, other materials are introduced or deposited onto its surface to form the composite.^67^ To fabricate the *ex situ* composite, various methods can be utilized. For example, metal nanoparticles or metal oxide precursors can be deposited onto the surface of pre-prepared g-C_3_N_4_ through methods like impregnation, photo-deposition, or chemical reduction. Carbon-based materials, such as graphenes or carbon nanotubes, can also be integrated with pre-formed g-C_3_N_4_ through solution mixing or deposition techniques. *Ex situ* crystallization offers advantages such as precise control over the loading amount and distribution of the additional material. It allows for flexibility in choosing the post-treatment conditions for efficient deposition or integration of the composite components, resulting in improved photocatalytic performance. The choice between *in situ* and *ex situ* crystallization depends on the specific composite design, the compatibility of the materials, and the desired properties. *In situ* crystallization allows for simultaneous formation of the g-C_3_N_4_ composite during the polymerization process, while *ex situ* crystallization offers flexibility in introducing and controlling the deposition of other materials onto pre-formed g-C_3_N_4_.^68^

### 2.3. Modification of g-C_3_N_4_ by metal-deposition

**Fig. 2:**
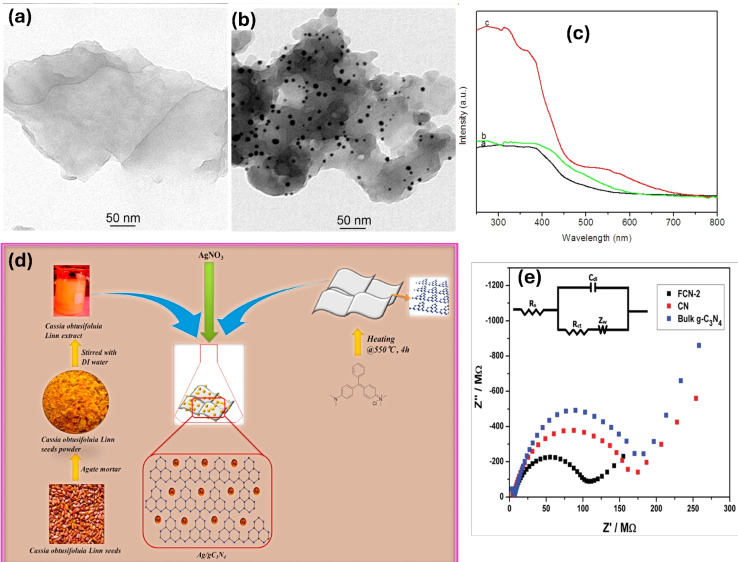
Tem image of (a) g-C_3_N_4_, (b) Au/g-C_3_N_4_, (c) DRS data of g-C_3_N_4_ nanosheets, bulk g-C_3_N_4_, and AuNP/g-C_3_N_4_ nanohybrids reprinted with the permission of ref. 72, copyright 2024, American Chemical Society; (d) synthesis of Ag/g-C_3_N_4_*via* green route, reprinted with the permission of ref. 73, copyright 2024, Elsevier; and (e) EIS of the g-C_3_N_4_, and pure and Fe-doped g-C_3_N_4_ nanosheets, reprinted with the permission of ref. 74, copyright 2024, RSC.

Metal deposition involves the introduction of metal nanoparticles or tiny thin films onto the surface of g-C_3_N_4_ through various deposition techniques, such as physical vapor deposition or chemical methods (*e.g.*, impregnation, electrochemical deposition).^69^ In this process, the metal species are not incorporated into the lattice structure of g-C_3_N_4_ but rather exist as separate entities on the surface. The incorporation of metals onto g-C_3_N_4_ as a composite photocatalyst offers critical prospects for improving its light absorption, charge separation, catalytic activity, and overall photocatalytic performance. The localized surface plasmon resonances, catalytic properties, and synergistic effects of noble metals contribute to the enhanced efficiency and selectivity of photocatalytic reactions. For instance, a facile immobilization of noble metals (Ag, Au, and Pd) onto g-C_3_N_4_ using a simple ultrasonication technique was described.^70^ In this method, g-C_3_N_4_ (0.5 g) was dispersed in DI water through ultrasonication for 1 hour. The metal precursor was then mixed with the previous suspension, followed by reduction using NaBH_4_ with continuous stirring for 1 hour. After noble metals' deposition, XRD examination showed a modest drop in the diffraction intensity of the g-C_3_N_4_ (100) plane. This implies that the presence of metal atoms prevented the formation of g-C_3_N_4_ crystals.^70^ Furthermore, Ag/g-C_3_N_4_ photocatalyst was synthesized by using an infrared-assisted heating strategy to deposit AgNO_3_ salt onto the g-C_3_N_4_. The presence of Ag nanoparticles on the surface of g-C_3_N_4_ facilitates the capture of electrons generated by g-C_3_N_4_ and their subsequent utilization in degrading methyl orange or producing H_2_ from H^+^.^71^ In another investigation, researchers employed ultrasonication-assisted liquid exfoliation to create g-C_3_N_4_ nanosheets from bulk g-C_3_N_4_.^72^ After that Au was deposited on g-C_3_N_4_*via* green photoreduction of Au(iii). TEM analysis verified the good exfoliation of bulk g-C_3_N_4_ (Fig. 2a). However, numerous Au NPs ranging from 5 to 20 nm were formed on the nanosheets, as depicted in (Fig. 2b). Additionally, DRS results demonstrated that the Au NPs/g-C_3_N_4_ composite exhibited an absorption peak at 550 nm, indicative of the surface plasmon resonance band specific to colloidal gold (Fig. 2c). Hence, the presence of Au NPs served as electron sinks, facilitating the separation of photogenerated electron/hole pairs.^72^ Moreover, Ag NPs/g-C_3_N_4_ composite was synthesized using an environmentally friendly chemical approach, as depicted in (Fig. 2d).^73^ The deposition of Ag NPs onto the g-C_3_N_4_ surface resulted in a slight reduction in the BET surface area, as shown in (Fig. 2d). XPS analysis further confirmed the existence of metallic silver on the g-C_3_N_4_ surface. Furthermore, chemical impregnation of single Pd atoms onto g-C_3_N_4_ enhanced its photocatalytic activity.^75^ The presence of single Pd atoms and their coordination structure in the composite were confirmed using HAADF-STEM (high-angle annular dark-field scanning transmission electron microscopy) and XAFS (X-ray absorption fine structure) analyses. The powerful interaction between the Pd- and surrounding N-atoms facilitated the production of photogenerated electrons, leading to the promotion of the photocatalytic performance of the composite.^75^ However, the noble metal's cost prevents its extensive use in real applications. Studies have been performed on various transition metals, including Fe, Cu, W, Zn, Mo, Zr, *etc.*^76–80^ For example, the incorporation of cobalt into g-C_3_N_4_ thorough a one-step thermal polycondensation approach suppressed the growth of the g-C_3_N_4_ crystals and resulted in a larger specific surface area with the formation of abundant Co–N_*x*_ active sites.^81^ It Also reduced the band gap energy and facilitated more efficient separation of photogenerated electrons and holes.^81^ Furthermore, the Fe/g-C_3_N_4_ composites were fabricated with various initial concentrations of FeCl_3_, resulting in samples labeled FCN-0.5, FCN-1, FCN-2, and FCN-3 representing 0.5%, 1%, 2%, and 3% Fe, respectively.^74^ The DRS revealed an enhanced visible-light range absorption and a redshift for Fe/g-C_3_N_4_ composites. As the Fe content increased, the optical band gap gradually shifted to lower energy, indicating the incorporation of Fe ions into the g-C_3_N_4_ lattice and altering its electronic structure. This redshift in absorption promoted the production of more electron–hole pairs under sunlight, ultimately enhancing the photocatalytic features. Additionally, the Nyquist plots illustrated clear differences in the semicircle diameter between bulk g-C_3_N_4_, pure g-C_3_N_4_, and FCN-2 nanosheets, with the FCN-2 nanosheets displaying a significantly smaller semicircle diameter compared to the others (Fig. 2e).^74^ Moreover, the Co/gC_3_N_4_ composite was fabricated through an *in situ* calcination strategy.^82^ Initially, 30 g of melamine was mixed with 50 mL of DI water. Subsequently, Co(NO_3_)_2_ was added to the suspension under sonication for 10 minutes, maintaining a weight ratio of 30 : 0.5. The resulting mixture was then calcined in a Muffle furnace at 550 °C for 1 hour at a heating rate of 10 °C min^−1^.^82^ Co/g-C_3_N_4_ had a surface area of 25.6 m^2^ g^−1^, featuring a larger amount of mesopores compared to g-C_3_N_4_ (surface area: 18.2 m^2^ g^−1^). The SEM image showed a mixed morphology in Co/g-C_3_N_4_, consisting of cobalt oxide grains with an irregular polygonal crystal shape and g-C_3_N_4_ sheets.

### 2.4. Modification of g-C_3_N_4_ by non-metallic and metallic doping

Doping involves introducing dopant into the lattice structure of g-C_3_N_4_ by substituting carbon or nitrogen atoms with dopant atoms. This process modifies the electronic structure and properties of g-C_3_N_4_ by altering the band structure, charge carrier mobility, and recombination rates. Non-metal and metal doping are the two primary types of elemental doping of g-C_3_N_4_. Non-metal doping has gained significant attention as a means to preserve the metal-free property of g-C_3_N_4_. Non-metals possess high ionization energies and electronegativities, allowing them to form covalent bonds by gaining electrons during reactions with other compounds.^83–86^ This characteristic makes non-metals a suitable option for doping g-C_3_N_4_, as they do not introduce metal ions with varying chemical states, which could be affected by thermal variations. Various non-metal dopants, including phosphorus, sulphur, carbon, nitrogen, oxygen, boron, and halogens, have been extensively investigated for their efficacy in doping g-C_3_N_4_.^87–89^

A facile method was employed to synthesize metal-free boron and oxygen-doped g-C_3_N_4_ with carbon vacancy.^90^ In this method, a mixture of g-C_3_N_4_ and varying amounts of H_3_BO_3_ (1%, 2.5%, 5%, and 10%) was ground and transferred to a crucible for calcination at 500 °C for 2 hours. The resulting B and O doped g-C_3_N_4_ exhibited distinct morphological characteristics compared to pristine g-C_3_N_4_, featuring loose and irregular tissue-like structures. SEM images revealed that the B and O dopants caused a modification in the morphology by dividing the bulk layers of g-C_3_N_4_ into smaller layers.^90^

Phosphorus-doped g-C_3_N_4_ was fabricated *via* a simple poly-condensation strategy using dicyandiamide (or cyanoguanidine) as the precursor and 1-butyl-3-methylimidazolium hexafluorophosphate as the phosphorus source.^91^ The hexafluorophosphate ions reacted with amine groups upon raising the temperature, incorporating phosphorus into the C–N framework. Analysis confirmed the formation of P–N bonds, with phosphorus likely substituting corner or bay carbon positions. Even at low doping levels, the electronic structure of g-C_3_N_4_ was significantly altered, leading to reduced optical band gap energy and increased electrical conductivity.^91^ Furthermore, P-doped g-C_3_N_4_ was synthesized *via* a thermal polymerization method, where the P atoms were successfully introduced into the g-C_3_N_4_ lattice, resulting in modified electronic properties and improved suppressions of charge carrier recombination.^92^ Moreover, a co-condensation approach, without the use of templates, was followed to synthesize P-doped g-C_3_N_4_ nanoflowers with in-plane mesopores, where the introduced phosphorus species exhibited strong chemical bonding with neighboring carbon and nitrogen atoms, leading to a forced planar coordination within the carbon nitride framework.^93^

Furthermore, a single-pot pyrolysis method was employed to synthesize sulfur-doped graphitic carbon nitride porous rods (S-pg-C_3_N_4_) by heating a complex of melamine and trithiocyanuric acid at various temperatures.^94^ The characterization results demonstrated that S-pg-C_3_N_4_ exhibited a porous rod structure with a significantly higher surface area (ranging from 20 to 52 m^2^ g^−1^) when compared to bulk g-C_3_N_4_. Additionally, it was observed that the surface area of the S-pg-C_3_N_4_ samples increased as the heating temperature was raised.^94^ On the other hand, the synthesis of oxygen-doped g-C_3_N_4_ using a facile H_2_O_2_ hydrothermal method was reported.^95^ XPS analysis revealed the successful doping of oxygen into the g-C_3_N_4_ lattice, resulting in the formation of N–C–O bonds, where oxygen atoms were directly bonded to sp^2^-hybridized carbon. Notably, the oxygen doping induced a downshift of the conduction band (CB) minimum by 0.21 eV without altering the valence band (VB) maximum. This oxygen doping-induced modulation of the electronic and band structure of g-C_3_N_4_ and led to various beneficial effects, including an increase in visible light absorption, extended surface area and enhanced photogenerated separation efficiency.^95^ Otherwise, using a hydrothermal synthesis, sulfur fluoride-doped carbon nitride (F-SCN) was effectively synthesized.^96^ The incorporation of fluorine and sulfur into the carbon nitride lattice resulted in a notable improvement in the photocatalytic performance by enhancing the separation of electron–hole pairs and facilitating efficient charge transfer.^96^

On the other hand, the g-C_3_N_4_ structure has been modified *via* metal doping.^97–100^ For example, mesoporous graphitic-carbon-nitride nanosheets doped with zinc ions (Zn-mpg-C_3_N_4_) were reported.^101^ The surface area and porosity of g-C_3_N_4_ were improved by PEG-1500, whereas the electrical features of the g-C_3_N_4_ increased when zinc was incorporated into the g-C_3_N_4_ structure.

### 2.5. Modification of g-C_3_N_4_ by creating heterojunctions

Heterojunctions in g-C_3_N_4_-based photocatalysts can be classified into several types based on their structural configurations and electronic band alignments, each offering unique advantages and functionalities for photocatalytic applications. Heterojunctions are typically formed by hybridizing g-C_3_N_4_ with other materials, *e.g.*, semiconductors or carbon materials, in a composite form. When these materials are nearby in a heterojunction, they maintain their distinct crystal structures and electrical properties. Different types of heterojunctions, such as Type-I, Type-II, p–n junctions, and Z and S schemes, can be used to create these connections.

#### 2.5.1. Modification by creating Type-I and Type-II heterojunctions

The synergistic combination of g-C_3_N_4_ with another photocatalyst can give rise to Type I and Type II heterojunctions, which exhibit fascinating electrochemical and optical properties.^102^ In Type I heterojunctions, the semiconductor with the wider band gap can promote efficient charge separation and migration. Specifically, when illuminated, electron–hole pairs can traverse from the VB and CB of the wider band gap semiconductor to the partner semiconductor, leading to enhanced photocatalytic performances.^102,103^ Furthermore, redox processes take place on the photocatalyst with a lower redox potential, modulating the overall photocatalytic activity. This complex interplay between different semiconductors and their band gaps exemplifies the potential for advanced applications in photocatalysis. For instance, the creation of customizable heterojunction structures composed of (CoO_*x*_) encapsulated within g-C_3_N_4_ using a straightforward one-pot technique under various annealing environments was demonstrated.^103^ A Type I heterojunction incorporating Co_3_O_4_/g-C_3_N_4_ nanotubes was established in an air setting, resulting in the aggregation of Co_3_O_4_ ranging from 20 to 80 nm on the nanotube surface. Another study reported the formation of type I and type II g-C_3_N_4_/g-C_3_N_4_ heterostructures for the removal of ppb-level NO in air.^102^ The research findings highlight the enhanced photocatalytic activity and stability of the g-C_3_N_4_-based heterostructures compared to pristine g-C_3_N_4_ alone. The improved performance can be attributed to the promoted charge separation within the heterostructures, leading to more efficient utilization of light energy and enhanced photocatalytic efficiency in NO removal.

Conversely, misalignment of the conduction and valence band boundaries among the two materials results in the creation of Type II heterojunctions, where the two semiconductors are interfaced while one semiconductor has a lower conduction band and the other has a higher valence band. An inherent electric field that is generated by the energy level movement at the interface may facilitate charge separation and boost charge migration across the junction. The CB potential of g-C_3_N_4_ typically around −1.1 eV, significantly lower than that of many other photocatalysts. Consequently, when exposed to irritation, e^−^ excited in the CB of g-C_3_N_4_ can swiftly move to the CB of a secondary photocatalyst with a greater potential. In parallel, the generated holes will move in the opposite direction. The creation of a Type II junction allows for the spatial separation of photogenerated electrons and holes, which prevents them from recombining and allows them to participate in desired redox reactions efficiently. This separation of charges leads to an increased lifetime of the charge carriers and enhances the photocatalytic activity of the system. Moreover, the band alignment in Type II heterojunctions can promote interfacial charge transfer processes, such as electron or hole transfer from one component to another, further improving the overall photocatalytic efficiency. This synergistic effect between different semiconductor materials in the heterojunction structure enables better utilization of solar energy and enhances the photocatalytic performance of g-C_3_N_4_-based systems. This phenomenon can be validated through specific analytical techniques like steady-state/time-resolved photoluminescence (PL) spectra, photocurrent measurements, and EIS measurements. Different types of semiconductor substances have been used in combination with g-C_3_N_4_ to create Type II heterojunctions to reduce the recombination of the generated charges, such as TiO_2_, ZnO, Fe_2_O_3_, MoO_3_, WO_3_, ZnTe, CdS, MoS_2_, ZnIn_2_S_4_, Bi_2_WO_6_, and others.^104–107^ For instance, various hierarchical heterojunctions of Bi_*x*_O_*y*_I_*z*_/g-C_3_N_4_, such as g-C_3_N_4_/BiOI, g-C_3_N_4_/Bi_4_O_5_I_2_, and g-C_3_N_4_/Bi_5_O_7_I have been successfully developed.^108^ The g-C_3_N_4_/BiOI is synthesized using a direct precipitation method, while g-C_3_N_4_/Bi_4_O_5_I_2_ and g-C_3_N_4_/Bi_5_O_7_I are obtained through *in situ* calcination transformation of g-C_3_N_4_/BiOI at different temperatures. The g-C_3_N_4_/BiOI and g-C_3_N_4_/Bi_4_O_5_I_2_ heterojunctions are classified as Type-I, while g-C_3_N_4_/Bi_5_O_7_I is categorized as a Type-II heterojunction. Notably, g-C_3_N_4_/Bi_5_O_7_I exhibited significantly improved performance compared to g-C_3_N_4_/BiOI and g-C_3_N_4_/Bi_4_O_5_I_2_. The promoted activity of g-C_3_N_4_/Bi_5_O_7_I can be attributed to its surface area, promote charge separation and transfer performance, and robust charge carrier density resulting from the formation of a Type-II heterojunction.

#### 2.5.2. Modification by creating p–n heterojunctions

The formation of a p–n heterojunction involves combining two different semiconductors with p-type and n-type electronic structures. This arrangement leads to a built-in electric field at the interface, which can promote charge separation and migration, thereby improving the photocatalytic performance of the material. g-C_3_N_4_ behaves as an n-type owing to the –NH/NH_2_ groups as electron donors present in its structure. Constructing a p–n heterojunction promotes the separation of electron–hole pairs. The Fermi level of a p-type (EF,p) is near its VB, while that of an n-type (EF,n) is close to its CB. When p-type and n-type contact, electrons transfer from the n- to p-type owing to the Fermi level offset. This results in a positively charged interface for the n-type semiconductor and a negatively charged interface for the p-type semiconductor, creating a built-in electric field at the contact interface. For instance, p–n CoFe_2_O_4_/g-C_3_N_4_ heterojunctions was created using a simple one-pot coprecipitation method.^109^ The development of the p–n heterojunction and the distinct structure of g-C_3_N_4_ facilitated charge separation and electron transfer, resulting in a remarkable enhancement in photocatalytic activity. The presence of an internal electric field at the junction boosted the accumulation of electrons and holes in the VB of g-C_3_N_4_ and the CB of CoFe_2_O_4_. This led to increased separation efficiency and a noticeable reduction in the recombination rate of electron–hole pairs. Other p–n heterojunctions, such as CuAl_2_O_4_/g-C_3_N_4_,^110^ BiOCl/g-C_3_N_4_,^111^ and MgIn_2_S_4_/g-C_3_N_4_ (ref. 112) have also been reported.

#### 2.5.3. Modification by creating Z-scheme and S-scheme heterojunctions

The Z-scheme heterojunctions were developed to address the limitations of conventional Type-II heterojunctions. In this arrangement, photogenerated electrons from photocatalyst II are transferred to the valence band (VB) of photocatalyst I. This process enhances the separation of charges in the semiconductor without altering the redox potential of the holes in the VB of photocatalyst II and the electrons in photocatalyst I. In the Z-scheme, the electrons and holes in the lower VB and higher CB levels can be utilized for generating reactive oxygen species (ROS). By maintaining the strong oxidative and reductive properties of the electrons and holes, this heterojunction is preferred over Type-II heterojunctions.^113^ However, some charge recombination between the lower VB and higher CB levels may still occur. In the direct Z-scheme, the transfer of electrons from one photocatalyst to another occurs directly through a physical contact or a solid-state interface between the two photocatalysts. This direct transfer of electrons enables efficient separation and utilization of charges for photocatalytic reactions. In the mediator Z-scheme, an additional mediator component is introduced between the two photocatalysts to facilitate the transfer of electrons. This mediator component acts as a shuttle, transferring electrons between the two photocatalysts, thus enabling efficient charge separation and reaction enhancement. The mediator Z-scheme provides flexibility in controlling and optimizing the electron transfer process in photocatalytic systems. For instance, 2D/2D Z-scheme BiOI-XBr/g-C_3_N_4_ with oxygen vacancies (OVs) was successfully fabricated.^114^ The introduction of OVs promoted visible-light absorption, acting as an electron mediator to accelerate the separation rate of photogenerated carriers in the Z-scheme. The optimal ratio of the heterostructures exhibited a high photodegradation activity for RhB, which was attributed to the synergistic effects of the 2D/2D Z-scheme heterostructure and OVs.

**Fig. 3:**
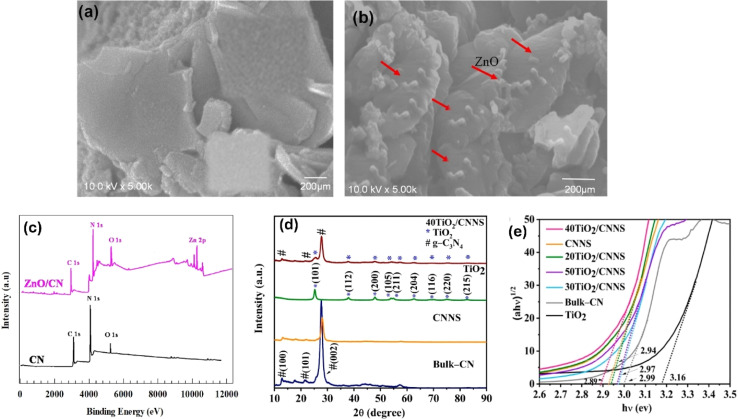
Surface morphology of (a) g-C_3_N_4_ and (b) ZnO/g-C_3_N_4_ and (c) XPS of g-C_3_N_4_ ZnO/g-C_3_N_4_, reprinted with the permission of ref. 119, copyright 2024, Elsevier; (d) PXRD patterns of bulk-g-C_3_N_4_ (CN), g-C_3_N_4_ nanosheets (CNNS), TiO_2_, and 40TiO_2_/CNNS, (e) Tauc plot displaying band gaps of g-C_3_N_4_, TiO_2_ and their composites.^120^

It is worthy to mention that metal oxides heterostructures can not only enhance the visible light absorption ability of g-C_3_N_4_ due to their unique band structures but also facilitate the separation and transfer of photogenerated electron–hole pairs, as well as improve the stability and reusability of g-C_3_N_4_ photocatalysts. The metal oxides act as protective layers, preventing the photocorrosion of g-C_3_N_4_ and enhancing its durability under harsh reaction conditions. This is particularly advantageous for long-term applications and practical implementation. The method used to incorporate the metal oxide into g-C_3_N_4_ can significantly impact the distribution and interaction between the two components, which ultimately affects the photocatalytic efficiency. For instance, TiO_2_ is a widely favored photocatalyst due to its excellent chemical stability, affordability, and suitable valence band (VB) and conduction band (CB) positions that facilitate redox reactions.^115,116^ Thus, a highly efficient heterojunction photocatalyst was developed by combining TiO_2_ nanotubes with g-C_3_N_4_ through a thermal deposition approach.^117^ In this process, a solution containing 100 mg of TiO_2_ nanotubes and 4 mg of g-C_3_N_4_ in 20 mL of distilled water was subjected to stirring at 80 °C for 6 hours. The HRTEM analysis confirmed the close attachment between TiO_2_ and g-C_3_N_4_, indicating a strong solid interaction and successful formation of the heterojunction.^117^ In a separate study, an S-scheme heterojunction of mesoporous/macro TiO_2_/g-C_3_N_4_ was fabricated using a straightforward chemical vapor deposition technique.^118^ The research revealed that by adjusting the melamine dosage, the microstructure of the samples could be readily controlled.^118^ Similarly, ZnO/g-C_3_N_4_ photocatalyst, consisting of ZnO loaded onto g-C_3_N_4_, was fabricated using an *ex situ* crystallization strategy.^119^ The images revealed that ZnO particles were present on the g-C_3_N_4_ layers, distinguishing it from pure g-C_3_N_4_ (Fig. 3a and b).^119^ XPS analysis confirmed the presence of Zn in the modified catalyst, indicating the successful combination of ZnO with g-C_3_N_4_ (Fig. 3c). Moreover, coral-like WO_3_/g-C_3_N_4_ were fabricated using a wet chemistry strategy, with different mass ratios of WO_3_ to g-C_3_N_4_ (1 : 1, 1 : 3, and 3 : 1). TEM images revealed that g-C_3_N_4_ appeared as ribbon-like sheets, surrounded by plate-like particles of WO_3_.^121^ The measurements of the crystallographic particle spacing between 0.20 and 0.39 nm suggest the existence of tiny crystalline zones in the g-C_3_N_4_ nanosheets. This close contact between g-C_3_N_4_ and WO_3_ facilitates the good separation of photo-excited carriers.^121^ Further, TiO_2_/g-C_3_N_4_ composites containing 20–50% TiO_2_ by weight were fabricated using a hydrothermal process by dispersing TiOSO_4_ in DI water, followed by the addition of g-C_3_N_4_ and ultrasonication for 30 minutes.^120^ The mixture was then heated in an autoclave at 180 °C for 4 hours. The resulting powder was dried at 65 °C. XRD patterns of the composites displayed peaks from both g-C_3_N_4_ and TiO_2_, with no shifting in the TiO_2_ peaks demonstrating that the TiO_2_ lattice structure was not impacted by the coupling with g-C_3_N_4_ (Fig. 3d). This lack of influence on the lattice structure is beneficial for photocatalytic activity. Moreover, among the composites, 40% TiO_2_/g-C_3_N_4_ had the lowest bandgap energy at 2.89 eV (Fig. 3e).^120^ In another study, MoO_3_/g-C_3_N_4_ was fabricated by combining 0.01 g of Mo_2_N with varying quantities of g-C_3_N_4_ and the resulting mixtures were subjected to calcination at 350 °C for 240 minutes.^122^

**Fig. 4:**
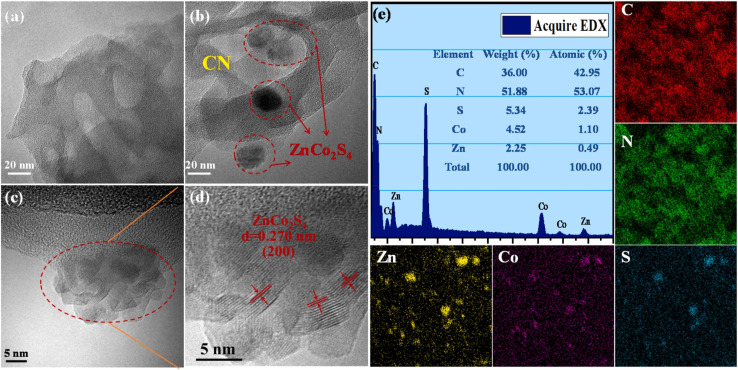
TEM images of (a) CN and (b) ZnCo_2_S_4_/CN, HRTEM images of (c–d) ZnCo_2_S_4_/CN, and (e) EDS spectrum of ZnCo_2_S_4_/CN and elemental mapping analysis, reprinted with the permission of ref. 132, copyright 2024, Elsevier.

Metal sulfides is another type of semiconductor materials, greatly enhancing the efficiency of photocatalysis.^123–126^ Metal sulfides possess band structures that meet the thermodynamic requirements for water splitting and exhibit improved responses to sunlight due to the formation of a less negatively charged valence band through the (S-3p) orbitals.^127^ These advantageous properties of metal sulfides significantly contribute to the superior photocatalytic performance of g-C_3_N_4_/metal sulfide heterojunction systems.^112,128^ The incorporation of metal sulfides allows for the creation of customizable band structures, thereby providing tangible benefits for the desired photocatalytic reaction. In a study, CdS/g-C_3_N_4_ core/shell nanowires were synthesized using a combination of solvothermal and chemisorption methods.^112^ Transmission electron microscopy (TEM) analysis revealed that g-C_3_N_4_ was effectively coated onto CdS nanowires, establishing intimate contact between the two materials. Additionally, the composite exhibited a higher surface area compared to pure CdS.^112^ In another investigation, a one-step solvothermal strategy was utilized to synthesize ultra-thin g-C_3_N_4_ (UCN) and incorporate NiS onto the surface of ZnIn_2_S_4_ (ZIS).^129^ The resulting ternary compound, NiS/ZnIS/UCN, was designed to possess dual great-speed charge transfer channels. By combining these materials, the composite achieved improved efficiency in H_2_ generation through enhanced charge transfer.^129^ It is evident from the TEM picture of NiS/ZIS/UCN that some NiS is loaded onto the surface of ZIS and UCN, implying that the heterojunction ternary compound of NiS/ZIS/UCN has been well constructed.^129^ In another work, a series of CoS_2_/g-C_3_N_4_ were fabricated through a photodeposition strategy.^130^ The size of the CoS_2_ species could be adjusted, ranging from single atom to nanometer scale, allowing for control over the photocatalytic features. The synthesis process involved mixing 20 mg of g-C_3_N_4_ with a solution containing 1 mL of 15.2 mg mL^−1^ thiourea aqueous solution, 1 mL of 5 mg mL^−1^ Co(CH_3_COO)_2_, 4 mL of ultrapure water, and 4 mL of absolute ethanol. The mixture was evacuated to remove air and then irradiated using a 300 W Xenon lamp to facilitate the deposition of CoS_2_ onto the g-C_3_N_4_ surface.^130^ In another work, a solvothermal approach was utilized to create a heterostructure photocatalyst made of g-C_3_N_4_/Bi_2_S_3_/CuS.^131^ Further, NiS/g-C_3_N_4_, CdS/g-C_3_N_4_, and CdS/NiS/g-C_3_N_4_ were created *via* a simple and dependable chemical deposition technique.^126^ In another study, g-C_3_N_4_ was coated with ternary NiCo_2_S_4_ using a solvent evaporation technique.^132^ Whereby, 30 mL of ethanol was used to dissolve sulphide nanoparticles and g-C_3_N_4_ nanosheets, and the mixture was then ultrasonicated for 30 minutes to create a homogenous suspension. Subsequently, the solvent evaporated at 70 °C, yielding a ZnCo_2_S_4_/g-C_3_N_4_ photocatalyst. The ZnCo_2_S_4_ nanoparticles, which are in very near proximity to the 2D g-C_3_N_4_ flakes, have a median size of around 20 nm, as determined by TEM investigation (Fig. 4a–d). Moreover, EDS analysis, on the other hand, confirmed that C, N, Zn, Co, and S coexist in the composite and that the atomic ratios of Zn, Co, and S are around 1 : 2 : 4, which is in agreement with the ZnCo_2_S_4_ theoretical chemical ratio (Fig. 4e).^132^

Pioneering studies constructed heterostructure with other different types of semiconductors, such as phosphides, carbonates, nitrides, halides, among others.^133–137^ For instance, Ag_2_CO_3_/g-C_3_N_4_ heterojunctions were fabricated using an ultrasonic method, where Ag_2_CO_3_ was sonochemically targeted and fixed to the g-C_3_N_4_ active centers.^135^

**Fig. 5:**
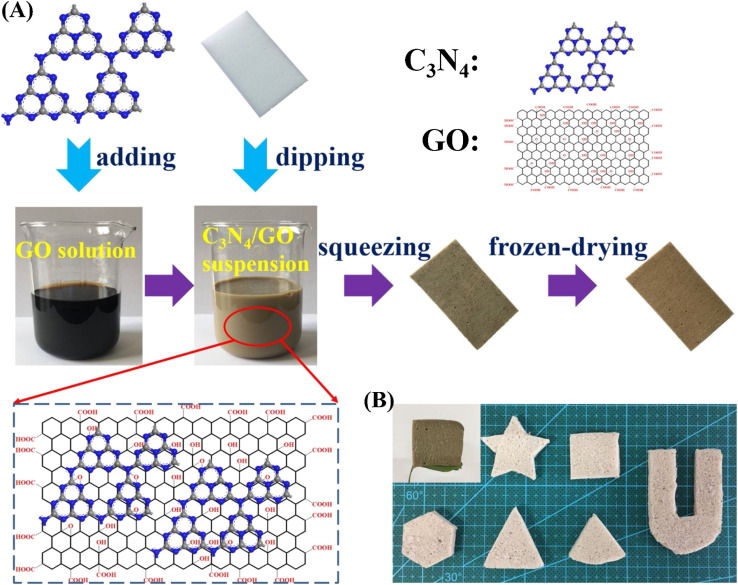
(a) Schematic illustration of the preparation of g-C_3_N_4_/GO-wrapped sponge; (B): image of different shapes of g-C_3_N_4_/GO-wrapped sponge, reprinted with the permission of ref. 150, copyright 2024, Elsevier.

**Fig. 6:**
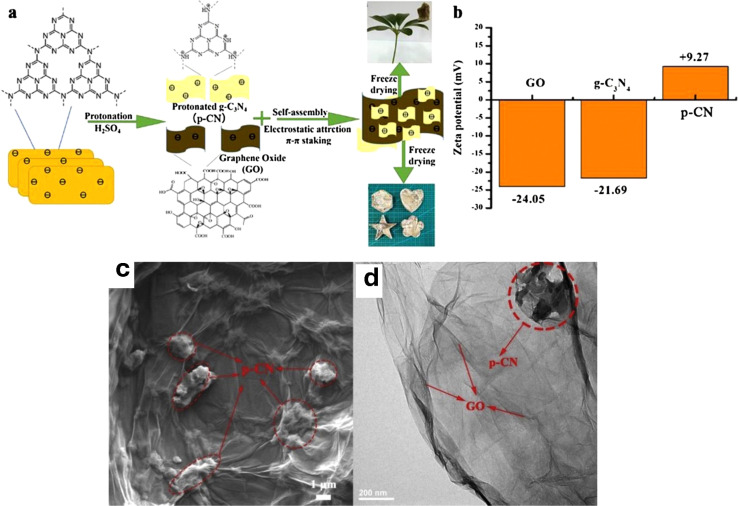
(a) Schematic of the fabrication of p-CN/GOA; (b) zeta potential of GO, g-C_3_N_4_ and p-CN, (c) the SEM of p-CN/GOA; (d) the TEM of p-CN/GOA, reprinted with the permission of ref. 152, copyright 2024, Elsevier.

**Fig. 7:**
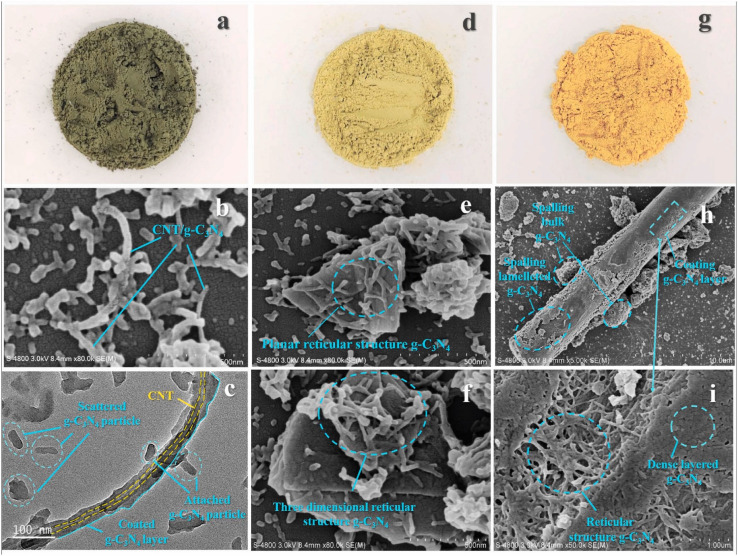
Macro shots of (a–c) CNT/g-C_3_N_4_, GN/g-C_3_N_4_ and CF/g-C_3_N_4_. SEM images of (d–f) CNT/g-C_3_N_4_, GN/g-C_3_N_4_ and CF/g-C_3_N_4_. TEM images of (g–i) CNT/g-C_3_N_4_, GN/g-C_3_N_4_ and CF/g-C_3_N_4_, reprinted with the permission of ref. 155, copyright 2024, Elsevier.

Carbon materials including graphene, carbon nanofibers, carbon nanodots, carbon nanotubes, and other forms of carbon materials, have gained significant attention for coupling with g-C_3_N_4_ in heterojunctions.^138–141^ Carbon materials possess symmetrical molecule arrangements with unique conjugated structures, offering superior photon excitation, high surface area, thermodynamic stability, and electron transmission.^142–145^ The creation of carbon-induced g-C_3_N_4_ photocatalysts presents a viable route for sustained improvements in photocatalytic technology as well as renewable carbon materials as an ecologically benign alternative to metal-based materials. Enhancement of photocatalytic processes has been obtained by modifications of carbon-induced g-C_3_N_4_ photocatalysts by several techniques such as junction interaction, surface reconstruction, cocatalyst effects, local electric modification, and more.^146–149^ For instance, g-C_3_N_4_/GO (graphene oxide)-wrapped melamine sponge (MS) monolith was developed through successful design and fabrication (Fig. 5).^150^ The g-C_3_N_4_ was uniformly distributed on the GO, ensuring efficient utilization of incident light and effective contact with pollutants. By acting as a bridge, GO facilitated the connection between the g-C_3_N_4_ and MS components. In another instance, g-C_3_N_4_/GO nanocomposite was synthesized by loading g-C_3_N_4_ onto GO using an electrostatic self-assembly approach.^151^ Furthermore, a unique protonated g-C_3_N_4_/GO aerogel (p-CN/GOA) was synthesized by a direct frozen-drying technique (Fig. 6a).^152^ The protonating treatment caused a significant change in the surface electric charge of g-C_3_N_4_, converting it from negative to positive (p-CN), which allowed for powerful self-assembly with the negative surface of GO. This assembly facilitated the transfer of photogenerated charge carriers. The stacking of p-CN blocks, which were several microns in size, were uniformly attached to the GO nanosheet due to the abundant surface functional groups of GO (Fig. 6c). While TEM confirmed the excellent loading of p-CN onto GO (Fig. 6d), providing further evidence of the combination between p-CN and GOA.^152^ In order to enhance the efficiency of underwater photocatalysis for g-C_3_N_4_, a composite consisting of g-C_3_N_4_ and carbon nanotubes (CNT) was fabricated using an *in situ* solvothermal approach.^153^ This composite had great surface area and improved light absorption capacity. The findings demonstrate that CNT and g-C_3_N_4_ exhibit good compatibility with each other. The g-C_3_N_4_ can grow directly on the surface of CNT, forming a stable composite structure.^153^ Another study used a straightforward water bath approach to construct g-C_3_N_4_ that had been enhanced with carbon nanotubes (CNTs).^154^ The morphological study showed that two materials were mixed together and that CNTs were wrapped in a lot of g-C_3_N_4_. This mixture promoted the movement of photogenerated electrons and aided in their separation efficiency.^154^ Further, carbon fibers (CF), graphene (GN), and CNTs were introduced to modify g-C_3_N_4_ through a solvothermal approach.^155^ The development morphology of the synthetic composites varied significantly depending on the utilized carbon substrate as shown in Fig. 7.^155^ The poor physicochemical features (*e.g.*, SBET, particle size, pore volume, adsorptive properties, … *etc.*), the limited photocatalytic catalytic activity, and stability and poor light-harvesting of pristine g-C_3_N_4_ are marginally boosted by proper modification and application of modified g-C_3_N_4_. The superior photocatalytic performance of modified g-C_3_N_4_ over pristine g-C_3_N_4_ is illustrated by various examples shown in Tables 1 and 2.

**Table 1:** Photocatalytic degradation performance of various g-C_3_N_4_ based binary photocatalysts^a^

Photocatalyst composite	Pollutant	Initial concentration (mg L^−1^)	Catalyst dose (mg)	Light source	Irradiation time (min)	Degradation (%)	Ref.
rGO–g-C_3_N_4_	RhB	10	8.0 mg	1000 W Xe lamp	100	75	156
MWCNTs–g-C_3_N_4_	MB	10	50 mg	300 W Xe lamp	180	100	157
	RhB				180	89.7	
	MO				180	84.5	
TiO_2_/g-C_3_N_4_	MB	20	100 mg	400 W Xe lamp	180	90	158
BN–g-C_3_N_4_	RhB	20	50 mg	300 W Xe lamp	120	98.0	159
	TC				60	79.7	
CQDs/g-C_3_N_4_	TC	10	500 mg	300 W XL	120	65	160
MoS_2_/g-C_3_N_4_	MB	5	NA	UV light	80	98.7	161
U doped C_3_N_4_	RhB	5		300 W XL	50	100	162
g-C_3_N_4_/ZnO	MB	10	50	Solar simulator	16	100	163
S-doped g-C_3_N_4_	MB	10	NA	100 W lamp	180	90	164
g-C_3_N_4_/CdWO_4_	TC	10	50	250 W Xe lamp	80	300	165
Ag-g-C_3_N_4_	MB	10	NA	200 W Xe	96	120	166
	CV				80		
	RhB				78		
Sm-g-C_3_N_4_	MY	20 mM	100	LED light	80	360	167
P-doped g-C_3_N_4_	RhB	20	20	Xe lamp	70	99.5	168
Fe-g-C_3_N_4_	RhB	10	20	300 W Xe lamp	45	90	169
BiOI exfoliated g-C_3_N_4_	TC	20	50	500 W xenon lamp	30	86	170
Ti_0.7_Sn_0.3_O_2_/g-C_3_N_4_	TC	20	25	1.5 W LED lamp	40	83	171
TiO_2_/g-C_3_N_4_	APAP	10	25	PLS-SXE300 Xe lamp (300 W)	45	96.7	172
C_3_N_4_–Ce_2_S_3_	ATZ	100	30	Xe lamp of 300 W (6280 lumens)	90	95	173
CN/MoO_3−*x*_	Phenol	50	NA	Full light, 300 W	60	98	174
CoO_*x*_/g-C_3_N_4_	MO	10	35	500 W Xenon lamp	180	92.0	175
	Phenol	10				49	
	norfloxacin	10				80	
Zn_3_V_2_O_8_/g-C_3_N_4_ Z	DZN	5	350	Visible light (180 mW cm^−2^)	60	95.2	176
BiVO_4_/g-C_3_N_4_	IMD	50	60	UV-C light (15 W m^−2^)	30	94.2%	177
Bi_2_WO_6_/g-C_3_N_4_	ATZ	800	200	500 W long-arc xenon lamp	180	99.9	178

a g-C_3_N_4_, graphitic carbon nitride; rGO, reduced graphene oxide; MWCNTs, multi-walled carbon nanotube; carbon dots (CDs) -BC, biochar; TC, tetracycline; RhB, rhodamine B, MB, Methylene blue; MO, methyl orange; TC, tetracycline; CV, crystal violet; DZN, diazinon; IMD, imidacloprid; atrazine, ATZ.

**Table 2:** Photocatalytic degradation performance of various g-C_3_N_4_ based ternary photocatalysts

Photocatalyst composite	Pollutant	Initial concentration (mg L^−1^)	Catalyst dose (mg)	Light source	Irradiation time (min)	Degradation (%)	Ref.
K-doped g-C_3_N_4_/BiOBr	RhB	20	50	500 W Xe	90	90	179
g-C_3_N_4_/CuO/ZnO	MB	10^−5^ mol L^−1^	50	Visible light	75	99	180
Ag/ZnO/S-g-C_3_N_4_	MB	10	10	Visible light (57–63 Klux)	60	98	181
Ag_10_-C_3_N_4_-NA_2_SO_4_	RhB	10	25	Visible light	50	96.5	182
g-C_3_N_4_/TiO_2_/carbon fiber	TC	10	25	350 W xenon lamp	90	99.9	183
Bi_2_O_2_CO/g-C_3_N_4_/Bi_2_O_3_	TC	10	10	Visible light (490–540) mW cm^−2^	60	80	184
WO@g-C_3_N_4_@MWCNTs	TC	20	20	Halogen lamp 500 W, 420 nm	120	79.5	185
AgPO_4_/g-C_3_N_4_/ZnO	TC	30	NA	45 W visible lamp	120	88.4	186
Bi_7_O_9_I_3_/g-C_3_N_4_/Bi_3_O_4_Cl	Phenol	10	50	NA	100	100	187
Ag@SrTiO_3_/g-C_3_N_4_	Dicofol	5	50	300 W Xe lamp	60	92.2%	188
BC-g-C_3_N_4_-MgO	Dinotefuran	10	100	(CEL-HXF300)	260	80.1	189
CDs@BiOI/g-C_3_N_4_	TC	20	NA	30 W LED	60	82.7%	190
MIL125(Ti)/g-C_3_N_4_/rGO	RhB	10	25	Fluorescent lamp (32 W)	120	98	191

## 3 Applications of g-C_3_N_4_ based nanocomposites

### 3.1. Applications in water treatment

#### 3.1.1. Photocatalytic degradation of organic pollutants

The environmental consequences of rapid industrial growth and diversification worldwide include the release of large volumes of contaminated water containing various organic pollutants, such as dyes, pesticides, pharmaceuticals, phenols, and others.^192–195^ As a solution to this issue, g-C_3_N_4_-based nanomaterials have emerged as highly researched photocatalysts for the treatment of wastewater contaminated with diverse pollutants. These nanomaterials offer numerous advantages, particularly effective adsorption and photocatalytic properties. In this context, we will delve deeper into the discussion of several g-C_3_N_4_-based composites employed for the removal of organic pollutants in wastewater treatment.

**Fig. 8:**
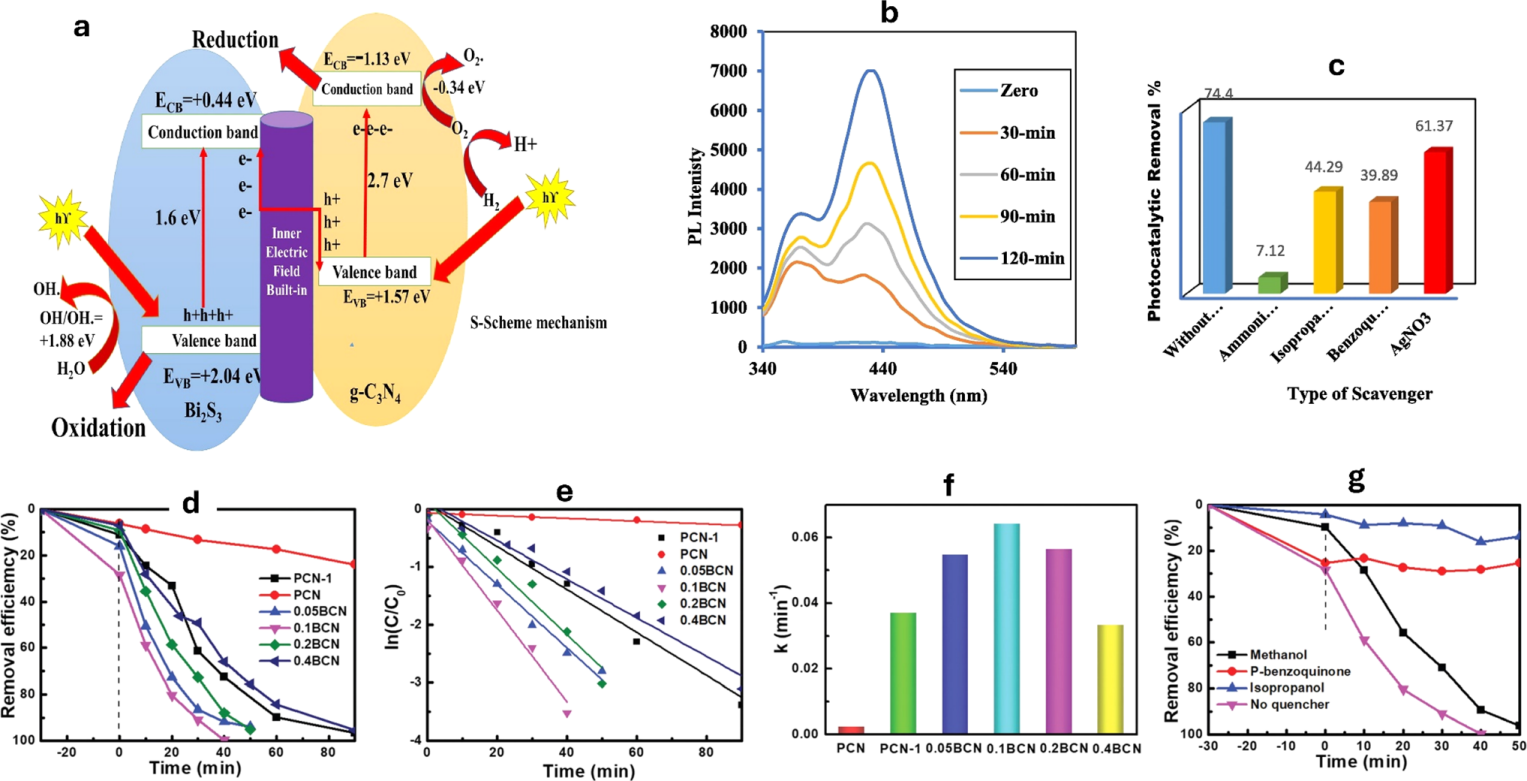
(a) An S-scheme for charge transfer between g-C_3_N_4_ and Bi_2_S_3_ in CNBiS10 catalyst, (b) PL of terephthalic acid over CNBiS_10_, and (c) effect of various scavengers on photocatalytic removal of terephthalic acid over CNBiS_10_ reprinted with the permission of ref. 196, copyright 2024, Elsevier; (d) degradation profiles of RhB catalyzed by polymeric carbon nitride (PCN) and Bi doped g-C_3_N_4_ at various ratios (BCN), (e) Pseudo first-order kinetics curves of RhB degradation, (f) apparent rate constant histogram of RhB, and (g) degradation of RhB with various radical quenchers reprinted with the permission of ref. 197, copyright 2024, RSC.

For instance, heterojunctions of Bi_2_S_3_/g-C_3_N_4_ with varying concentrations of Bi_2_S_3_ have been developed for the Rhodamine B (RhB) degradation under sunlight.^196^ The photocatalytic response is moved to the deep visible spectrum by depositing Bi_2_S_3_ on g-C_3_N_4_. When exposed to natural solar radiation, the rate of RhB dye breakdown on 10% Bi_2_S_3_/g-C_3_N_4_ is four times higher compared to bare g-C_3_N_4_ and Bi_2_S_3_ alone. This is explained by the fact that Bi_2_S_3_ nanoparticles extend optical reactivity under the whole range of natural sunlight, which lowers the rate at which hole–electron pairs recombine, promotes large charge-carrier movement, and ultimately raises photocatalytic efficiency. The decomposition of RhB is primarily impacted by positive holes, radical species, and superoxide radicals. The S-scheme mechanism described the movement of charge carriers (Fig. 8a), as revealed by terephthalic acid PL examinations and radical scavenging tests (Fig. 8b and c).^196^

Further, the degradation of methylene blue dye (MB) was carried out using MoO_3_/g-C_3_N_4_ heterojunction enhanced with biomass carbon dots. In comparison to bulk g-C_3_N_4_, pure MoO_3_, and pure carbon dots, the heterojunction demonstrated a better degradation rate of 67% throughout one hour of simulated sunlight irradiation.^198^ Under ideal compounding circumstances, the heterojunction between MoO_3_ and g-C_3_N_4_ was verified, resulting in an enhanced charge transfer rate at the interface.^198^

To enhance the photocatalytic activity of g-C_3_N_4_, the researchers loaded g-C_3_N_4_ with different magnesium salts.^199^ Among the various samples tested, the MgSO_4_-g-C_3_N_4_ composite exhibited the highest efficiency for photocatalytic degradation, achieving a photodynamic parameter of 26.36 × 10^−3^ min^−1^. Reactive substances including O_2_˙^−^, h^+^, and ˙OH that oxidized MB during the photocatalytic degradation process, where the ˙OH was the most contributing species.^199^ In another investigation, g-C_3_N_4_ was loaded with potassium salts such as KF, KCl, and KBr, resulting in the formation of KX-g-C_3_N_4_ (X = F, Cl, and Br).^200^ Remarkably, KF-g-C_3_N_4_ exhibited exceptional performance in the degradation of MB when exposed to visible light. Notably, KF-g-C_3_N_4_ effectively suppressed the recombination of holes and electrons, surpassing the photocatalytic activity of KCl-g-C_3_N_4_, KBr-g-C_3_N_4_, and pure g-C_3_N_4_ materials.^200^ On the other hand, bismuth/g-C_3_N_4_ nanotubes (BCN) with a porous structure having various bismuth fractions (0.05–0.40 g) were utilized for the RhB degradation of.^197^ The highest degradation efficiency was observed with the 0.1 BCN sample, which completely degraded RhB within 40 minutes (Fig. 8d). The degradation kinetics followed pseudo-first-order behavior (Fig. 8e), and the rate constant (*k* for 0.1 BCN was 0.0644 min^−1^), which was 26.8 times higher than that of pure g-C_3_N_4_ (PCN) (Fig. 8f), where the degradation was inhibited in the presence of isopropanol and *p*-benzoquinone (Fig. 8g).^197^ Furthermore, by adopting a straightforward impregnation technique, g-C_3_N_4_-TiO_2_ nanocomposites with varying weight proportions (1 : 3, 2 : 2, and 3 : 1) were produced. Under UV-visible illumination, the effectiveness of these nanocomposites in MB dye photocatalytic degradation was examined.^201^ When contrasted with virgin g-C_3_N_4_ and different weight percentages of g-C_3_N_4_/TiO_2_, the nanocomposite with a 3 : 1 weight ratio had the highest photocatalytic activity. Because there were fewer TiO_2_ nanoparticles deposited on the g-C_3_N_4_ nanosheets, the electron–hole pair transport features were improved, which increased the catalytic efficiency. The creation of a Z-scheme system between TiO_2_ and g-C_3_N_4_ explains the improved photocatalytic behavior.^201^

**Fig. 9:**
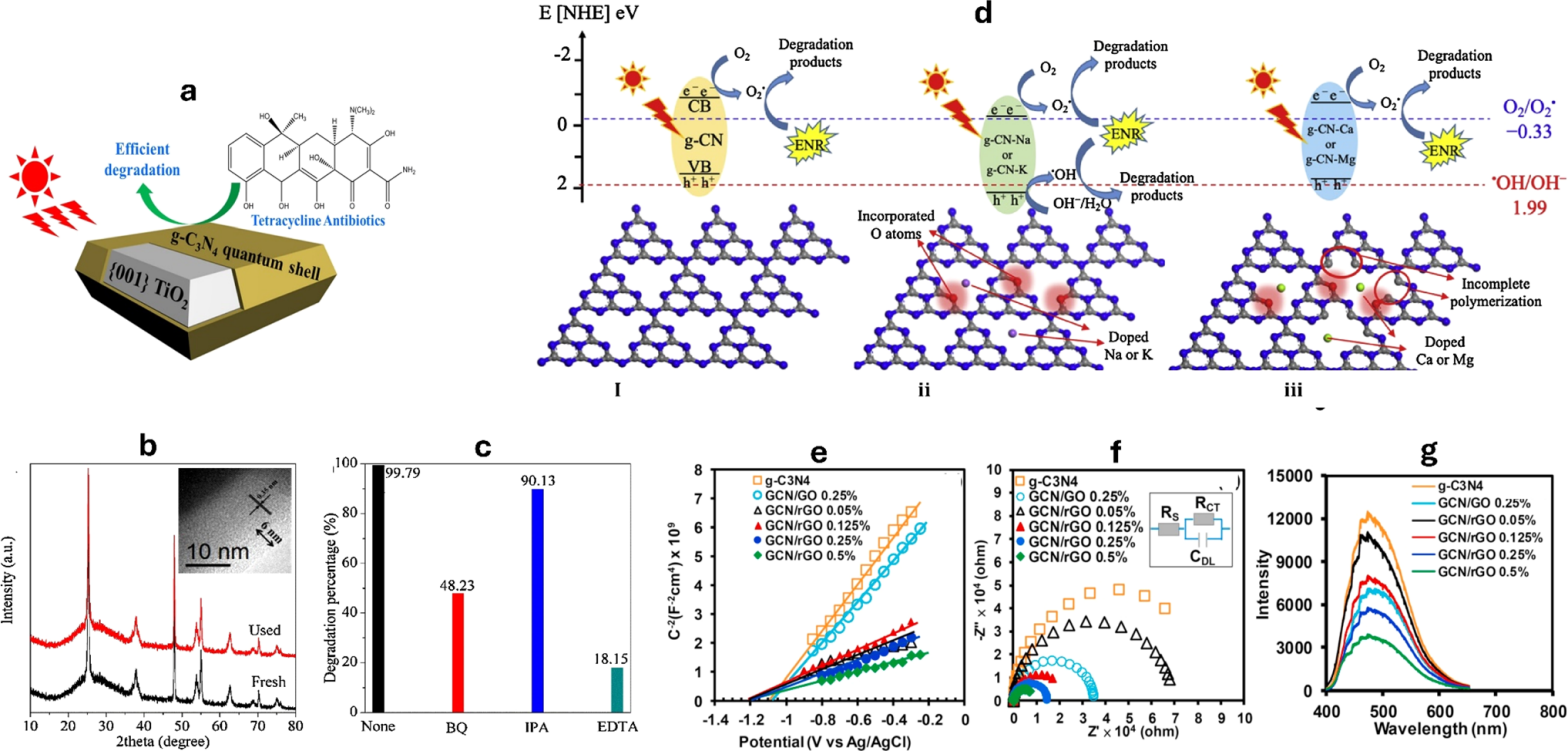
(a) Degradation of tetracycline by TiO_2_/g-C_3_N_4_ (TCN),^202^ (b) XRD patterns of TCN before and after TC degradation process, TEM image (inset) of the used TCN, (c) effect of different scavenger on TCN photocatalytic activity reprinted with the permission of ref. 202, copyright 2024, Elsevier; (d) degradation mechanism of enrofloxacin (ENR) by g-C_3_N_4_, in the absence and the presence of Na, K, Ca, and Mg dopants reprinted with the permission of ref. 203, copyright 2024, Elsevier; (e) EIS measurements presented as the Mott–Schottky plot,^204^ (f) the Nyquist plot, and (g) the photoluminescence spectra of bisphenol A photodegradation in the presence of rGO/g-C_3_N_4_ nanocomposites with different rGO ratios reprinted with the permission of ref. 204, copyright 2024, Elsevier.

In order to create the TiO_2_@g-C_3_N_4_ (TCN) core–shell quantum heterojunction, an effective way of polymerizing the quantum-thick g-C_3_N_4_ onto the surface of TiO_2_ with exposed facets was adopted and applied the obtained nanocomposite to the photocatalytic degradation of tetracycline (TC), as shown in Fig. 9a.^202,205^ The maximum rate of tetracycline degradation, exhibited using 100 mg TCN nanocomposite photocatalyst, was 2.2 mg min^−1^; that is 36% more than the rate observed in the TiO_2_/g-C_3_N_4_ random mixture (TCN(mix)), twice as high as TiO_2_, and 2.3 times higher than pure g-C_3_N_4_. The distinct advantages of the structure of the quantum-thick g-C_3_N_4_ shell, the abundance of readily accessible reaction sites, and the compact and consistent contact interface, are what make TCN more photocatalytically active. The notable improvement in the photocurrent responsiveness of TCN electrodes further supports efficient mobility of electrons among TiO_2_ and g-C_3_N_4_. The catalyst's stability was verified by TEM analysis and XRD, as shown in Fig. 9b. The principal oxidant species for the successful photocatalytic process, according to the results, are h^+^ and ˙O_2_^−^, as shown in Fig. 9c.^202^ Furthermore, the researchers found that the improved catalytic activity of CuAl_2_O_4_/g-C_3_N_4_ for TC photodegradation is mainly due to the significant separation of charge carriers, as shown by the transient photocurrent response.^110^ Moreover, the use of g-C_3_N_4_ loaded various metals (Na, K, Ca, Mg) has been studied for the degradation of enrofloxacin (ENR).^203^ The presence of oxygen atoms in the g-C_3_N_4_ nanocomposites has been confirmed through XPS, TEM, and FTIR analysis. These added metals, combined with the oxygen atoms, have altered the electronic structures and morphology of the g-C_3_N_4_, resulting in reduced charge recombination and improved light absorption. As a result, g-C_3_N_4_–Na and g-C_3_N_4_–K produced both hydroxyl radicals and superoxide, while g-C_3_N_4_, g-C_3_N_4_–Ca, and g-C_3_N_4_–Mg only produced superoxide radicals (Fig. 9d). In another study, the integration of graphene onto the edges of g-C_3_N_4_ enhanced the absorption of photons with energies below the intrinsic bandgap.^206^ This integration resulted in a broad-spectrum-driven response and facilitated near-field electron transfer. The strong π-conjugated bond-stitched nanostructures between graphene and g-C_3_N_4_ were found to effectively capture adsorbed oxygen molecules, leading to the production of ˙O_2_^−^, promoting the interaction between pollutant molecules and the photocatalyst NPs.^206^ Additionally, the incorporation of reduced graphene oxide (rGO) into g-C_3_N_4_ greatly enhanced the photocatalytic activity of bisphenol A (BPA) approximately three times at neutral pH to give 99% removal within 60 minutes.^204^ The synthesized rGO/g-C_3_N_4_ nanocomposite exhibited increased electrical conductivity and improved surface area, leading to enhanced separation of electron–hole pairs, as shown in Fig. 9e and g. The positioning of heterocyclic nitrogen p_*z*_ orbitals in g-C_3_N_4_ was shifted after decorating with rGO, facilitating the polarization of charge distribution, and resulting in the formation of active holes that boosted the BPA degradation.^204^

#### 3.1.2. Effects of operational parameters

Developing effective and long-lasting photocatalytic systems requires a thorough examination of the impact of operating parameters on the photocatalytic breakdown of organic pollutants utilizing composites based on g-C_3_N_4_. To optimize the process, it is essential to comprehend how pH, temperature, coexisting pollutants, light intensity, catalyst dose, and pollutant concentration interact. However, it is crucial to remember that depending on the particular pollutant and photocatalyst under investigation, these characteristics may have different effects.

**Fig. 10:**
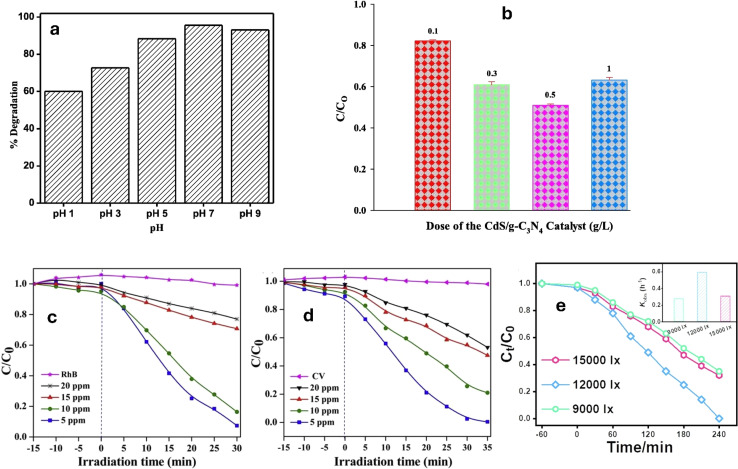
(a) Effect of pH on degradation of RhB dye reprinted with the permission of ref. 213, copyright 2024, Elsevier, (b) the effect of catalyst mass on degradation of MO dye, reprinted with the permission of ref. 214, copyright 2024, Elsevier; (c and d) effect of initial RhB and CV dye concertation on the degradation performance reprinted with the permission of ref. 215, copyright 2024, Elsevier; (e) effect of light intensity on sulfamethoxazole removal, reprinted with the permission of ref. 216, copyright 2024, Elsevier.

One of the key parameters to consider is the pH of the reaction medium. pH influences not only the adsorption capacity of the catalyst but also the protolytic equilibria involving the catalyst and the pollutant, as well as the pollutant's solubility.^207,208^ These factors can significantly affect the surface charge of the catalyst and the pollutant molecules, thereby impacting their interaction and subsequent degradation efficiency.^209^ Therefore, determining the optimum pH range is essential to maximize the photocatalytic performance. However, it should be noted that pH optimization is highly dependent on the specific pollutant and composite being used, as different materials may exhibit different pH sensitivities. The photocatalyst shows positive/negative zeta potentials depending on pH, demonstrating that its surface charge varies significantly with the solution's pH.^210^ For instance, the researchers investigated the pH impact on the degradation of RhB and MO dyes, using g-C_3_N_4_@NiAl LDH catalyst.^211^ They found that the catalyst had a point of zero charge (PZC) value of 6.6 where the highest efficiency for degrading MO occurred at a pH of 3, while RhB degradation was most effective at a pH of 10. Since RhB is a positively charged dye, it experiences repulsion when it approaches the positive surface of the catalyst in the presence of free H^+^ ions, leading to lower degradation at pH 3 compared to neutral or basic conditions. Similarly, MO degradation was reduced under basic circumstances by competition and repulsion among the OH^−^ anions and the anionic MO moieties for adsorption on the photocatalyst.^211^ Additionally, in the photodegradation of trimethoprim (TMP), peroxymonosulfate (PMS) can be activated by Fe-g-C_3_N_4_ with various compositions.^212^ Thus, it was shown that 0.2% Fe-g-C_3_N_4_/2 wt% rGO/PMS greatly increased the TMP degradation rate in the acidic environment (pH = 3), from 61.4% at pH = 6 to almost 100%. On the other hand, at basic pH levels, where TMP existed primarily as an anionic species, the repulsion among the Fe-doped g-C_3_N_4_/rGO composites and TMP hindered its degradation, leading to lower performance.^212^ Furthermore, g-C_3_N_4_/TiO_2_ (PZC = 6.0) exhibited the highest effectiveness in basic and neutral pH conditions, which promoted the interaction between the cationic RhB molecules and the catalyst's negatively charged surface functional moieties at pH > 6, leading to improved photodegradation of RhB, as shown in Fig. 10a.^213^ Conversely, g-C_3_N_4_/rGO exhibited pH-sensitive photocatalytic performance toward the photocatalytic degradation of the Rh Cationic dye, with a significantly greater rate of photodegradation at low acidity levels (pH = 1.98).^217^ The rate of RhB photodegradation dropped markedly as pH increased and reached almost zero at pH ≥ 7. This pH sensitive behavior was attributed to the promoted electron-transfer, at lower pH, between RhB, H^+^, and rGO that acted as a good platform for transferring e^−^ through its atomic sheets.^217^

The weight or loading amount of the catalyst material can impact various aspects of the photocatalytic process, ultimately affecting the degradation efficiency. One of the key aspects influenced by the weight of the catalyst is surface area.^218^ Increasing the weight of the catalyst generally leads to an increase in the available active surface area for pollutant adsorption and subsequent reaction.^209,219,220^ This can be beneficial as it provides more sites for catalytic activity, allowing for a higher number of reactive species to be generated. Consequently, the degradation rate of the organic pollutant may increase with increasing catalyst dose. However, it is important to note that there is an optimum weight or loading amount beyond which further increases may not result in proportional enhancements in degradation efficiency.^221^ This is because excessively high loadings can lead to aggregation or agglomeration of the catalyst particles, reducing the accessible surface area and hindering the photocatalytic process.^209^ Moreover, high-weight loadings can also cause light scattering or absorption, limiting the penetration of photons and reducing the overall photocatalytic activity. For instance, the photocatalytic performance of TiO_2_/g-C_3_N_4_ improved with increasing catalyst doses until the optimal dose was reached due to the enhancement in the available active sites.^222^ Moreover, the photocatalytic degradation efficiency of MO dye increased with the CdS/g-C_3_N_4_ mass; however, beyond the optimal mass the catalyst particles tended to aggregate, resulting in increased light scattering and lowered overall effective surface area, as well as reduced catalytic activity.^214^ The results presented in Fig. 10b indicate that the optimal dose of CdS/g-C_3_N_4_ for achieving the highest photodegradation of MO is 0.5 g L^−1^. A similar trend was also observed for the degradation RB in the presence of g-C_3_N_4_/CdO photocatalyst.^223^

Increasing the initial pollutant's concentration can lead to a greater number of pollutant molecules available for adsorption onto the catalyst surface.^195,224^ This can result in improved initial degradation rates, as more pollutant molecules can interact with the generated reactive radicals. Higher pollutant concentrations can also lead to an increased chance of collisions between the target molecules and the photocatalyst, enhancing the overall degradation efficiency. However, it is important to note that there is an optimum concentration beyond which increasing the initial pollutant concentration may not lead to further enhancements in photocatalytic activity. This is primarily due to two factors. Firstly, at high concentrations, the adsorption sites on the catalyst surface may become saturated, hindering further adsorption of the pollutant molecules. This can limit the availability of reactive radicals and decrease the overall degradation efficiency. Secondly, high concentrations of pollutant molecules in the reaction medium can absorb or even scatter the incident light, preventing it from reaching the photocatalyst surfaces effectively.^225^ Consequently, the generation of electron–hole pairs and the subsequent reactions may be limited, resulting in reduced photocatalytic activity. For instance, the rate of degradation of the rhodamine B and crystal violet (CV) dyes by the zeolite nanorods decorated g-C_3_N_4_ nanosheets (H-ZSM-5/g-C_3_N_4_) was demonstrated in Fig. 10c and d, illustrating the impact of varying starting dye concentrations.^215^ In this case, a pseudo-first-order (PFO) kinetic model explained the dye elimination process. The degradation rate was low at a high concentration (20 ppm) owing to light being impeded from reaching the active sites by the high chromaticity dye molecules present in considerable quantities. Other researchers reported a reduction in the dye degradation at higher concentrations owing to competition among hydroxyl ions and organic substances on active sites as well as the distracted light before reaching the catalyst surface.^223^ Similarly, in studying the effect of loading ZnO/g-C_3_N_4_ nanocomposites with aluminum, magnesium, nickel, copper, and silver, on the degradation rate of 50–300 mg L^−1^ Eriochrome Black T dye (EBT), the results showed a decreased dye degradation efficiency at higher concentrations of the EBT dye.^226^

The light intensity plays a significant role in photocatalytic degradation processes as it directly affects the absorption of photons by the catalyst. Higher light intensities provide a greater number of photons, leading to increased electron–hole pair generation and subsequent formation of reactive species, resulting in improved degradation rates.^68^ However, it is important to note that once a certain light intensity threshold is reached, further increases may not proportionally enhance photocatalytic activity. In fact, excessive light intensities can lead to increased energy consumption without providing substantial benefits. Thus, optimizing light intensity is crucial to achieve the optimal photocatalytic performance. Factors such as the source of light, the wavelength, and the type of catalyst used should all be considered when determining the ideal light intensity for a specific photocatalytic system. For instance, the photocatalytic degradation of sulfamethoxazole (SMX) using Fe-UCN's catalyst was greatly affected by the used light intensity. Under 9000, 12 000, and 15 000 lx of LED light intensity, the SMX % removals were 48%, 75%, and 53%, respectively, as shown in Fig. 10e.^216^ Therefore, while more intense light may provide the catalysts with photons for creating ˙OH and lower the pollutant's concentration, too much light may actually inhibit photocatalytic activity due to excessive electron consumption, resulting in the accumulation of extra holes on the catalysts, which hinders the photodegradation process.^216^

**Fig. 11:**
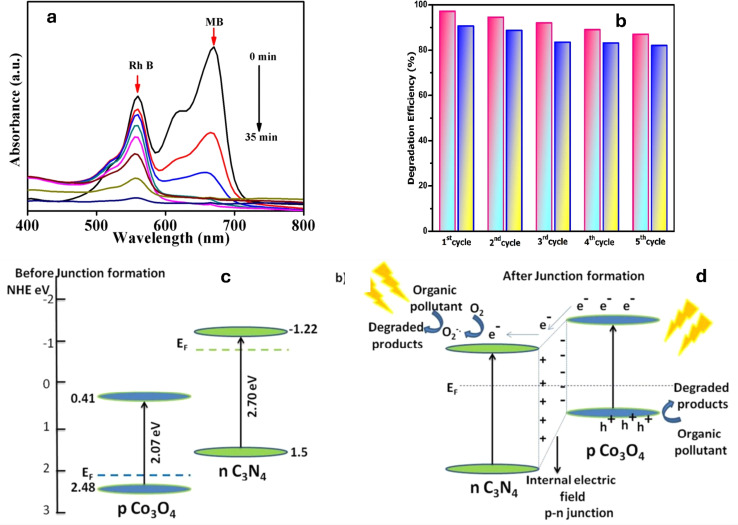
(a) UV absorption spectrum of Mixed dye (MB + RhB) by ZnFe_2_O_4_–CN,^228^ (b), recycling catalytic activity measurement for mixed pollutants,^229^ (c) band alignment of p-type Co_3_O_4_ and n-type C_3_N_4_ before junction formation and (d) band alignment and the photocatalytic mechanism of Co_3_O_4_–C_3_N_4_ p–n nano-heterojunctions, reprinted with the permission of ref. 230, copyright 2024, RSC.

The presence of multiple pollutants can lead to either synergistic or inhibitory effects on the degradation process. Synergistic effects occur when the presence of one pollutant enhances the degradation of another pollutant, due to the formation of reactive species or the modification of the degradation pathway. On the other hand, inhibitory effects can occur when the presence of one pollutant hinders the degradation of another pollutant, due to interactions between the pollutants that can compete for reactive species or affect the availability of active sites, thereby reducing the overall degradation efficiency. Therefore, it is crucial to consider the interactions between pollutants in a mixed system when evaluating degradation efficiency. For instance, pCN-N/ZIS Z-scheme heterojunction was evaluated for the synergistic photodegradation of metronidazole (MNZ) and methyl orange (MO).^227^ The combination of electron-donating groups on MO and MNZ molecules and electron traps on catalyst surfaces, which improves the catalyst's capacity to contact and adsorb pollutants and ultimately improves the catalytic degradation performance.^227^ Moreover, the degradation of a mixed MB and RhB dye solution was investigated using ZnFe_2_O_4_-g-C_3_N_4_ as the photocatalyst with the addition of H_2_O_2_ under sunlight illumination. The MB degradation rate was found to be much greater than that of RhB. As shown in Fig. 11a, after 35 minutes of exposure to sunlight, the maximum removal of MB was 100%, and in the presence of H_2_O_2_, the maximum removal of RhB was 92%.^228^ Similarly, the synergistic degradation efficiency of g-C_3_N_4_/α-Fe_2_O_3_ for the mixed RhB and MB solution was reported.^229^ At five cycles, the fabricated catalyst exhibits a high-performance, as shown in Fig. 11b. Conversely, Co_3_O_4_/g-C_3_N_4_ nano-heterojunctions were fabricated to degrade a mixture of TC antibiotic and MB dye pollutants, under solar irradiation. The researchers noticed that MB in the mixed solution showed an improved degradation rate (nearly 100% in 120 minutes) than when it was eliminated individually (90% in 120 minutes).^230^ When compared to the TC antibiotic's solo activity (97% in 180 minutes), the mixture's antibiotic degradation efficiency was slightly lower (78% in 180 minutes). The formation of intimate interfaces with enhanced photophysical properties was attributed to the band bending induced by the p–n nano-heterojunctions, as shown in Fig. 11c and d.^230^ The degradation efficiency of a g-C_3_N_4_-based Ce_2_O_3_/CuO (GCC) ternary nanocomposite was studied for mixed anionic metanil yellow (MY) and cationic MB dyes, under visible light exposure.^231^ Notably, the ternary GCC nanocomposite exhibited excellent performance, achieving high removal efficiencies for both MY and MB aqueous dyes (94.5% and 90.3%, respectively). This superior performance can be attributed to the optimal amounts of Ce_2_O_3_ and CuO present on the g-C_3_N_4_ surface, which facilitated the creation of heterojunction surfaces, thereby efficiently reducing the recombination rates of photo-excited charges.^231^

### 3.2. Hydrogen production

The production of hydrogen as a clean and sustainable energy source has gained significant attention in recent years. Several strategies were employed to enhance the photocatalytic activity of g-C_3_N_4_, which is an efficient photocatalyst that utilizes solar energy to split water and produce hydrogen. When excited by photons with energy equal to or higher than its bandgap, g-C_3_N_4_ can generate electron–hole pairs that can be involved in a series of reactions to produce hydrogen. The key steps involved in photocatalytic hydrogen production include light absorption, charge, separation, surface reactions and mass transfer.^232,233^

One common approach is the modification of g-C_3_N_4_ through metal co-catalyst decoration. For instance, platinum (Pt) nanoparticles can be loaded onto g-C_3_N_4_ to enhance the hydrogen evolution reaction (HER) kinetics by providing active sites for hydrogen formation. Other transition metals, such as nickel (Ni) and cobalt (Co), have also been utilized as co-catalysts due to their cost-effectiveness and abundance. Thus, the researchers created a combination of sulfidized bimetallic nickel and platinum decorated g-C_3_N_4_ with various Pt masses for the production of H_2_ using visible light. They found that the addition of the NiS_*x*_ electron acceptor in the S-PtNi_*X*_/g-C_3_N_4_ catalyst resulted in improved performance compared to catalysts without it, such as PtS_*x*_ or Sulfidized-g-C_3_N_4_.^234^ The existence of PtNi_*X*_ assisted in the correct transmission of charges. The impressive photocatalytic activity of the S-PtNi_*X*_/g-C_3_N_4_ catalyst, which achieved a rate of 4966 μmol g^−1^ h^−1^, can be attributed to the collaborative effect of NiS_*X*_ ability to accept electrons and PtNi_*X*_ superior charge transfer capabilities.^234^

Another study described single Pt atom co-catalysts embedded on g-C_3_N_4_*via* a procedure that involves two stages including incipient wetness impregnation and copolymerization.^235^ During a 4 hours period, the studies conducted with pure g-C_3_N_4_ exhibited minimal activity, generating around 12.7 μmol h^−1^ g^−1^. This suggests that subjecting g-C_3_N_4_ to visible light resulted in minimal photocatalytic efficiency. In contrast, the photocatalytic hydrogen evolution of g-C_3_N_4_ dramatically improved upon the adoption of 0.1–0.3 wt% of single Pt atoms as co-catalysts. The photocatalytic H_2_ evolution for Pt_0.1_-g-C_3_N_4_, Pt_0.2_-g-C_3_N_4_ and Pt_0.3_-g-C_3_N_4_ were about 1054.3, 4875.0 and 2932.8 μmol g^−1^ in 4 h, respectively. The highest rate of hydrogen generation was obtained with 0.2% Pt-based catalyst, due to its highest negative CB location and remarkable capacity to separate and transmit photogenerated charge carriers.^235^

Moreover, a heterojunction consisting of NiS grown on a 2D ultrathin g-C_3_N_4_ matrix was constructed for visible light-induced H_2_ generation.^236^ The presence of the NiS/g-C_3_N_4_ resulted in a synergistic impact, effectively enhancing the separation of photo-generated carriers and promoting interfacial charge transfer performance. The rate of H_2_ generation using the exfoliated NiS/g-C_3_N_4_ catalyst reached 4.2 μmol h^−1^ g^−1^, which is approximately 2.6 times higher compared to bulk C_3_N_4_/NiS.^236^ The creation of 0-D/2-D heterojunctions using g-C_3_N_4_ nanosheets and polyfluorene dots (Pdots) (Pdots/g-C_3_N_4_) was investigated and showed a substantial rise in photocatalytic HER, reaching 929.3 μmol g^−1^ h^−1^ with an apparent quantum efficiency of 5.7% at 420 nm.^237^

The photocatalytic water-splitting capability of Ag_3_PO_4_/g-C_3_N_4_ has been studied.^238^ The nanocomposite band gap energy value of 2.90 eV, suggest that it may be a successful visible light-harvesting composite. According to the research, compared to the electrons in the CB (0.21 eV) of g-C_3_N_4_, the Ag_3_PO_4_ electrons in CB (−1.08 eV) showed more potential for reducing water and protons to form H_2_. Similarly, VB holes of g-C_3_N_4_ exhibited stronger oxidizing capabilities than those of Ag_3_PO_4_, resulting in the production of ·OH radicals. Ag_3_PO_4_/g-C_3_N_4_ composite showed an electron transformation mechanism that resulted in the production of a Z-scheme process, which is beneficial for water splitting to produce H_2_.^238^

Using Ti_3_C_2_ MXene as a precursor, carbon-doped TiO_2_ (C–TiO_2_) linked with g-C_3_N_4_ was synthesized.^239^ In comparison to pure TiO_2_ with average particle size of 25 nm (P25), the C–TiO_2_ exhibited a lowered bandgap of 2.94 eV, implying boosted visible light absorption with a redshifted absorption edge at 425 nm. As a result, the 10% C–TiO_2_/g-C_3_N_4_ catalyst produced hydrogen at a rate of 1409 μmol g^−1^ h^−1^ (*λ* > 420 nm) with enhanced activity ascribed to the creation of a Type II heterojunction, which enables optimum charge separation and increased accessorial surface area, offering extra reaction sites upon coupling with C–TiO_2_.^239^

Moreover, the researchers applied FeO_*x*_/g-C_3_N_4_ for the improved efficiency H_2_ evolution through water splitting.^240^ The optimized amount of FeO_*x*_ led to an impressive H_2_ evolution rate of 108 μmol h^−1^ that is 4.2 times higher than that of pristine g-C_3_N_4_. Numerous reasons, such as increased surface area, greater electron transfer ability, better visible light absorption, and superior charge carrier separation, are responsible for this improvement.^239^

Other researchers conducted a study on the fabrication of g-C_3_N_4_/CNTs for achieving high-efficiency H_2_ production.^241^ They incorporated different types of CNTs, including single-walled (SW), double-walled (D), and multi-walled (MW), to enhance the activity of g-C_3_N_4_-based photocatalysts. Enhanced production of photocatalytic hydrogen was seen when the amount of CNTs is low, leading to a boost in the stability and quantity of photogenerated charges. The improved electron transport from g-C_3_N_4_ to CNTs, which was particularly apparent in the case of SWCNTs, accounts for this improvement.^241^

Additionally, the S-scheme heterojunction of N-doped MoS_2_/S-doped g-C_3_N_4_ was successfully constructed using a straightforward one-step thermal polymerization approach.^242^ Following material optimization, the catalyst's photocatalytic hydrogen generation rate reached 658.5 μmol g^−1^ h^−1^. This was made possible by the boost in visible light absorption and photogenerated carrier separation yield caused by the S-scheme's design.

Further, a comprehensive investigation on the impact of three common transition metal phosphides (M_2_P, M = Fe, Co, and Ni) as cocatalysts in sulfur-doped g-C_3_N_4_ (S–CN) was investigated.^243^ The researchers utilized an ultrasound-assisted approach to create M_2_P/S–CN with similar load ratios, ensuring comparable crystallization levels and particle sizes. Ni_2_P/S–CN demonstrated the most rapid charge transfer and separation among the three phosphides, resulting in smaller photocatalytic overpotential. This remarkable performance yielded a rate of hydrogen generation that was comparable to that of Pt/S–CN catalysts and 22.7 times higher than that of bare S–CN.^243^

Otherwise, a simple wet-chemical fabrication approach was used to successfully produce a dual Z-scheme heterostructure of g-C_3_N_4_, PrFeO_3_, and Fe_2_O_3_.^244^ This cascade dual Z-scheme exhibited impressive production, generating 379.29 μmol g^−1^ h^−1^ under visible-light exposure. The inclusion of magnetic components in the heterostructure facilitated the easy separation of the catalyst and enabled its reusability. Additionally, RuNi/g-C_3_N_4_ catalysts doped with 2D bimetallic RuNi alloys were created using the solvothermal deposition approach involving various Ru ratios. The catalyst sample having 2.3 wt% Ru revealed the greatest hydrogen evolution, reaching 35 100 μmol g^−1^ h^−1^, surpassing the performance of the Pt/g-C_3_N_4_ photo-catalyst.^245^Table 3 shows the photocatalytic H_2_-evolution performance characteristics of representative g-C_3_N_4_-based photocatalysts.

**Table 3:** Photocatalytic H_2_ evolution performance of various g-C_3_N_4_-based photocatalysts

Photocatalyst composite	Sacrificial agent	Catalyst dose (mg)	Light source	H_2_ evolution rate	Ref.
Graphene with 1% wt. and g-C_3_N_4_	Methanol	80 mg	350 W Xe arc lamp	451μmol h^−1^ g^−1^	246
AgIO_4_/g-C_3_N_4_	Methanol	0.1 g	Solar simulator	23 mmol h^−1^ g^−1^	247
BiVO_3_/g-C_3_N_4_	Methanol	0.05 g	350 W Xenon	6.8 mmol g^−1^ h^−1^	248
C_60_/g-C_3_N_4_/graphene	TEOA	100 mg	5 W light-emitting diode (LED) irradiation	545 μmol h^−1^ g^−1^	249
Graphene/ZnIn_2_S_4_/g-C_3_N_4_	—	5 mg	Solar light	545 μmol h^−1^ g^−1^	250
TiO_2_/g-C_3_N_4_	Methanol	0.1 g	Xenon lamp of 350 W	4.9 mmol g^−1^ h^−1^	222
SnO_2_/g-C_3_N_4_	Methanol	0.1 g	Xenon lamp of 350 W	6.56 mmol g^−1^ h^−1^	251
g-C_3_N_4_/0.25% RGO/3% NiS	TEOA	50 mg	300 W Xe arc lamp	393 μmol h^−1^ g^−1^	252
Cu_2_O@g-C_3_N_4_	TEOA	0.3 g	300 W xenon lamp	265 μmol h^−1^ g^−1^	253
TiO_2_–g-C_3_N_4_	Methanol	0.1 g	Xenon lamp	35.44 μmol h^−1^ g^−1^	254
g-C_3_N_4_/WO_3_	—	50 mg	300 W Xe lamp	982 μmol h^−1^ g^−1^	255

### 3.3. Carbon dioxide reduction

**Fig. 12:**
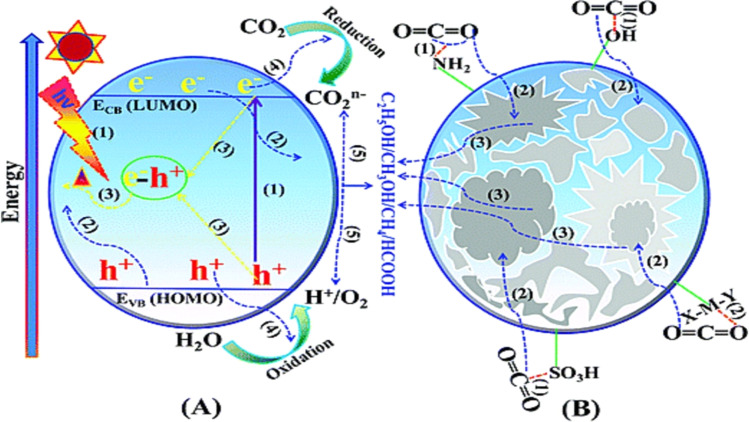
General steps involved in CO_2_ photoreduction coupled with water oxidation: (A) optoelectronic: (1) e^−^–h^+^ generation, (2) charge migration to the surface, (3) e^−^–h^+^ recombination, (4) CO_2_ photoreduction, (B) physicochemical: (1) CO_2_ adsorption, (2) CO_2_ activation, and (3) product desorption.^257^

**Fig. 13:**
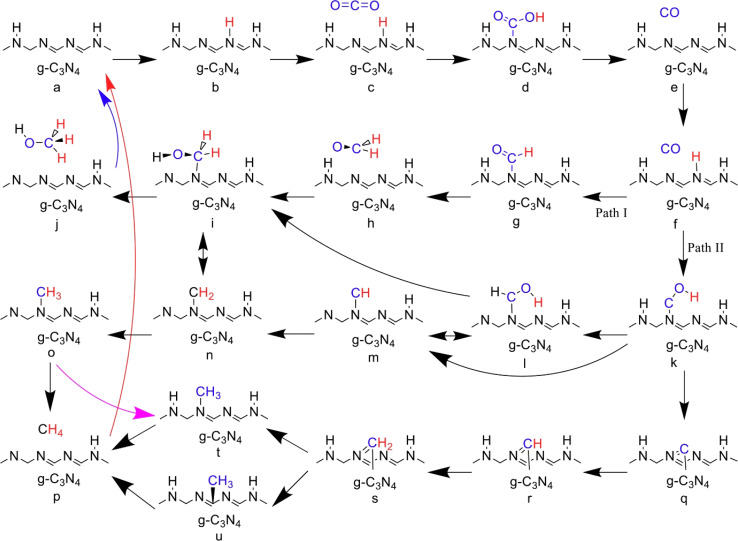
Proposed reaction pathway for CO_2_ reduction to methanol and methane on the surface of g-C_3_N_4,_ reprinted with the permission of ref. 258, copyright 2024, Elsevier.

Applying g-C_3_N_4_-based nanocomposites for CO_2_ reduction holds significant promise for addressing the global challenge of climate change by transforming CO_2_ emissions into valuable products, such as methane, methanol, and hydrocarbons. The photocatalytic activity of g-C_3_N_4_-based composites is attributed to their unique structure and composition, which facilitate the absorption of light and generation of reactive species for CO_2_ sequestration, contributing to the reduction of greenhouse gas emissions and the development of a circular carbon economy.^256^Fig. 12 depicts the basic steps involved in CO_2_ photoreduction involving surface and optoelectronic properties.^257^

g-C_3_N_4_ based photocatalysts play a robust role in the process of CO_2_ photoreduction through their optoelectronic and physicochemical features. These catalysts expose active sites on their surfaces where CO_2_ adsorption and activation take place. Therefore, when designing C_3_N_4_-based photocatalysts, it is essential to prioritize factors such as efficient visible light absorption, promote surface area, quick electron transfer to the catalyst surface, exposure of functional groups, minimized recombination rate, and a robust redox potential value. Fig. 13 shows how CO_2_ is transformed into methane and methanol on the surfaces of g-C_3_N_4_.^258^ The process starts by capturing and activating CO_2_ when two electrons are generated.^257^ Then, an intermediate called COOH* is produced, which eventually converts into CO. The hydrogenation of CO* into COH* or CHO* is a significant step in CO_2_ reduction.^258^ For instance, the Ag_3_PO_4_@g-C_3_N_4_ hybrid promoted the photocatalytic reduction of CO_2_.^259^ This was achieved by forming a heterojunction structure between Ag_3_PO_4_ and g-C_3_N_4_, which promoted the CO_2_ reduction activity through a Z-scheme mechanism that facilitated the charge separation phenomena. When exposed to simulated sunlight, the optimized Ag_3_PO_4_@g-C_3_N_4_ hybrid demonstrated a robust CO_2_ conversion rate of 57.5 μmol h^−1^ g_cat_^−1^, surpassing the rates of pure g-C_3_N_4_ and P25 catalysts by 6.1 and 10.4 times, respectively. Further, graphene-supported 1D nano-arrays of crystalline carbon nitride (1D-CCN) heterojunction was developed and demonstrated promoted interface charge transfer, facilitated light absorption, and promoted CO_2_ capture capabilities.^260^ Furthermore, the 1D-CCN demonstrated a 44% selectivity for CO_2_ over N_2_, with isosteric heat adsorption of 55.2 kJ mol^−1^ for CO.

**Fig. 14:**
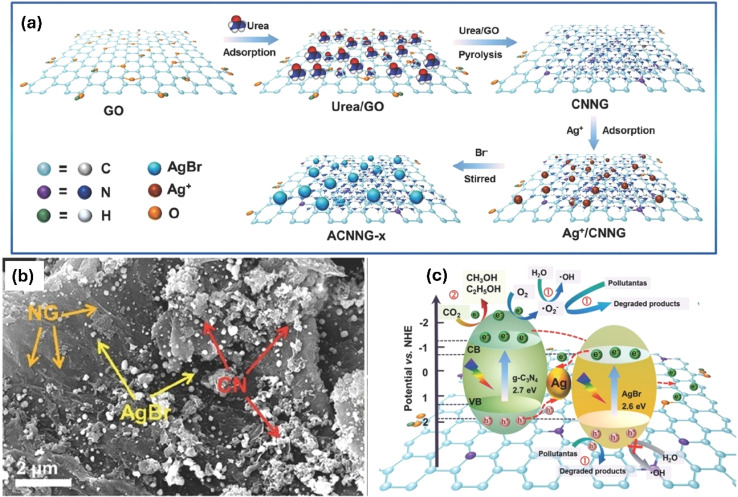
(a) Synthesis process of ACNNG-*x* hybrid, and (b) its SEM image, and (c) a mechanism for CO_2_ reduction by the hybrid nanocomposites, reprinted with the permission of ref. 264, copyright 2024, Elsevier.

Researchers have introduced B, S, Mo, O, and P heteroatoms into g-C_3_N_4_ to promote its performance in CO_2_ photoreduction.^79,260–263^ Among these dopants, O- and P-doped g-C_3_N_4_ demonstrated robust conversion capabilities compared to pure g-C_3_N_4_. Additionally, S-doping and creating N-vacancies can introduce impurities in the conduction band position of g-C_3_N_4_, expanding light absorption to longer wavelengths and minimizing recombination rates. Moreover, the researchers created ternary hybrids (ACNNG-*x*) by combining AgBr with g-C_3_N_4_-modified nitrogen-doped graphene (NG) in various ratios.^264^ These catalysts were employed for reducing CO_2_ using visible light. The process of making the composite and SEM image of the optimized ternary hybrid are displayed in Fig. 14a and b, respectively. The optimized ternary composite demonstrated promising CO_2_ reduction rates of 105.89 μmol g^−1^ for methanol and 256.45 μmol g^−1^ for ethanol. A proposed mechanism for the process is presented in Fig. 14c. Similarly, g-C_3_N_4_/NaNbO_3_ nanowires were synthesized for CO_2_ reduction.^265^

Enhancing the overall system performance by modifying g-C_3_N_4_ with a component for CO_2_ adsorption has proven effective. For instance, g-C_3_N_4_ combined with a cobalt-containing zeolitic imidazole framework (Co-ZIF-9), demonstrated high CO_2_ adsorption capacity (2.7 mmol g^−1^) and a significant microporous surface area (1607 m^2^ g^−1^), facilitating CO_2_ capture and concentration in its pores.^266^ The addition of bipyridine as an electron mediator allowed photoexcited electrons to transfer from g-C_3_N_4_ to Co-ZIF-9 for CO_2_ reduction, as shown in a PL quenching study. In this system, CO was the primary product, achieving a quantum efficiency of 0.9% without the need for a cocatalyst.^266^ Moreover, g-C_3_N_4_/Bi_2_WO_6_ hybrid was hydrothermally fabricated to selectively convert CO_2_ to CO through photoreduction.^267^ The hybrid demonstrated a visible-light CO generation rate of 5.19 mmol g^−1^ h^−1^, surpassing that of g-C_3_N_4_ alone. The hybrid's improved photocatalytic activity was attributed to effective charge separation and transfer following a Z-scheme mechanism.^267^

### 3.4. Hydrogen peroxide production

Hydrogen peroxide (H_2_O_2_) production using g-C_3_N_4_-based photocatalysts is a promising approach that has gained significant attention in recent years. The photocatalytic strategy for generation H_2_O_2_ typically involves two major approaches: the reduction of O_2_ and the oxidation of H_2_O. The reduction of O_2_ can occur through a one-step, two-electron process or a two-step, one-electron process. The oxidation of H_2_O, on the other hand, occurs in a single step, with photogenerated holes driving the reaction. However, the direct oxidation of H_2_O for H_2_O_2_ production is challenging due to the robust thermodynamics involved and the tendency of H_2_O_2_ to act as a scavenger for the photogenerated holes at high oxidation potentials.^268–271^

**Fig. 15:**
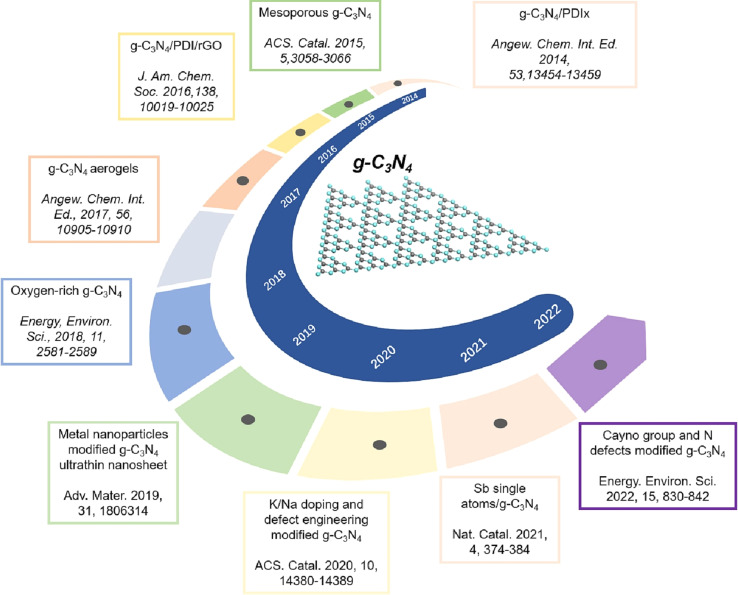
A summary of recent H_2_O_2_-generation methods based on g-C_3_N_4_ photocatalysts.^269^

The photocatalytic production of H_2_O_2_ using g-C_3_N_4_-based composite photocatalysts typically involves the reduction of oxygen (O_2_) to H_2_O_2_ using the photogenerated electrons in the conduction band of the composite. The key to efficient H_2_O_2_ production lies in the ability of the composite to efficiently absorb visible light and facilitate the separation of photogenerated electron–hole pairs. Recent progress of g-C_3_N_4_-based catalyst for H_2_O_2_ production is shown in Fig. 15.^269^

One of the key factors that influence the H_2_O_2_ production efficiency of g-C_3_N_4_-based composite photocatalysts is the design of the heterojunction interface. A widely studied g-C_3_N_4_-based composite photocatalyst for H_2_O_2_ production is the g-C_3_N_4_/TiO_2_ system.^272^ Where *n* S-scheme heterojunction promoted the light absorption and the separation of photogenerated charges, resulting in enhanced photocatalytic performance and improved H_2_O_2_ production.^273^ Additionally, pairing of g-C_3_N_4_ with other materials, such as GO and metal–organic frameworks (MOFs) further improved the H_2_O_2_ production efficiency.^274,275^

In the case of g-C_3_N_4_/GO composites, the GO acts as an efficient electron acceptor, facilitating the separation of photogenerated electron–hole pairs in the composite.^191^ The high specific surface area and excellent electrical conductivity of GO contribute to the improved H_2_O_2_ production efficiency. Similarly, the integration of g-C_3_N_4_ with MOFs can provide a high surface area and tunable pore structure, enhancing the adsorption of reactants and the photocatalytic H_2_O_2_ production.^276^

The choice of materials and their relative positioning within the composite can synergistically impact the light absorption, charge separation and transfer processes and the overall catalytic activity. Computational studies using density functional theory (DFT) calculations have provided valuable insights into the electronic structure, band alignment, and charge carrier dynamics at the g-heterostructure interfaces.^276,277^ DFT calculations and experimental data attributed the boosted photocatalytic activity of the modified heterostructure to the positively charged MOF sheet interlayer, and the coupling between MOF nanosheet, g-C_3_N_4_, and CuO that can enrich ions, electrons, and molecules and obstruct holes to greatly boost the rapid separation of photogenerated carriers from g-C_3_N_4_ and/or CuO, and the reactants' adsorption.^276^ Thus, incorporating suitable metal active centers into the g-C_3_N_4_ framework is an effective approach to promote the activity and selectivity of the oxygen reduction reaction (O_2_RR).^278^ The adsorption of O_2_ on the metal surface can occur in three different configurations: Griffiths-type (side-on), Pauling-type (end-on), and Yeager-type (side-on).^269^ For instance, the researchers developed a novel Sb-single-atom photocatalyst (Sb-SAPC) doped g-C_3_N_4_ that exhibited exceptional performance, generating H_2_O_2_ at 12.4 mg L^−1^, 248 times higher than pristine g-C_3_N_4_.^279^ The enhanced activity of Sb-SAPC-g-C_3_N_4_ is attributed to the Sb-SAPC sites that facilitated O_2_ adsorption and activation, where the accumulation of photogenerated holes at neighboring N-atoms near Sb sites promotes the oxygen evolution reaction (OER) for O_2_ production. The Sb–OOH intermediates suggest a direct one-step, two-electron reduction pathway for H_2_O_2_ generation.^279^

Forming a heterojunction structure is a successful approach to addressing the challenge of charge carrier recombination in pristine g-C_3_N_4_. This is because the difference in Fermi level between g-C_3_N_4_ and the coupled co-catalysts drives the photogenerated charge carriers to migrate between the two components. For instance, a 2D/2D heterojunction composed of ZnIn_2_S_4_ and g-C_3_N_4_ (ZIS/CN) was prepared employing a simple oil bath heating approach.^280^ The obtained data demonstrated that the H_2_O_2_ production proceeded through a 2-electron oxygen reduction (2e^−^ O_2_RR), reflecting a robust selectivity towards H_2_O_2_ generation. The promoted photocatalytic performance was attributed to the synergistic impact of intimate interfacial contact. In another study, an oxygen-doped g-C_3_N_4_ modified g-C_3_N_4_/TiO_2_ (OCN@CNT-2) hybrid system was constructed through an electrostatic self-assembly approach, where a double Z-scheme architecture was formed within the target OCN@CNT-2 composite.^272^ This unique heterojunction promotes the charge separation under the influence of an internal electric field. As a result, after 60 minutes, the system was able to achieve a remarkably high H_2_O_2_ yield of up to 133.04 μmol L^−1^.

The produced H_2_O_2_ can find various applications, including water purification, disinfection, and oxidation processes. Ongoing research aims to further improve the H_2_O_2_ production efficiency, stability, and scalability of g-C_3_N_4_-based composite photocatalysts, paving the way for their practical implementation in large-scale H_2_O_2_ production systems.

## 4 Conclusion and prospective

In conclusion, this comprehensive review article has covered various aspects of g-C_3_N_4_ based nanocomposites, including their synthesis and characterization methods, their application in the removal of organic pollutants and hydrogen production, and the factors influencing their photocatalytic activities. Through the incorporation of dopants, metal deposition, metal chalcogenide semiconductors, and carbon materials, these nanocomposites have exhibited remarkable photocatalytic capabilities with potential for real-world environmental remediation and energy production. The synthesis and characterization techniques discussed in this article have provided valuable insights into enhancing the performance and stability of g-C_3_N_4_-based composites. The introduction of dopants and metal deposition, as well as metal chalcogenide semiconductors have enabled the modification of the band structure and surface properties, thereby improving the separation and transfer of photogenerated charge carriers. The incorporation of carbon materials, such as graphene or carbon nanotubes, has contributed to the enhancement of photocatalytic activity by increasing the surface area and facilitating electron transfer. The photocatalytic degradation of various organic pollutants, including dyes, pesticides, and pharmaceutical compounds, has been effectively achieved using g-C_3_N_4_ based composites. Additionally, the production of hydrogen as a clean and sustainable energy source has been successfully demonstrated through photocatalytic water splitting. The investigation of factors affecting the photocatalytic process has deepened our understanding of the mechanisms involved and has highlighted the important working factors such as catalyst dose, pH, and light intensity. This knowledge can be utilized to optimize the design of g-C_3_N_4_ based nanocomposites, tailoring them for specific applications and improving their overall performance and efficiency.

Looking to the future, there are several exciting prospectives for further development in the field.

1. The scale-up of synthesis methods and the development of cost-effective production techniques are essential for the practical application of g-C_3_N_4_-based composites. Efforts should also be made to evaluate their long-term stability and recyclability to ensure their viability for laboratory, pilot-plant and large-scale implementation with the involvement of engineering and chemistry disciplines.

2. In the pursuit of constructing novel g-C_3_N_4_-based photocatalysts, there is a need for template-free and environmentally friendly synthetic approaches that can yield unique structures and exceptional intrinsic properties. However, the current methods of modifying these photocatalysts have certain limitations. Some of the selected composite materials contain expensive and environmentally detrimental elements. Achieving precise chemical doping of g-C_3_N_4_ is a difficult task that often results in the introduction of impurities. Furthermore, the available techniques for controlling the structure of g-C_3_N_4_ are relatively limited and have only minimal effects. Additionally, achieving precise control over the microstructure of these photocatalysts remains a challenging endeavor.

3. More detailed and specific reporting is needed to elucidate the synergistic effects that occur among the individual materials in complex heterostructures.

4. While there is a theoretical understanding of the charge transfer and separation pathways, further experimental evidence is necessary to validate these photochemical mechanisms and establish effective photocatalytic systems on a larger scale.

5. In the realm of photocatalytic degradation, it is crucial to address the simultaneous degradation several pollutants present in real wastewater using g-C_3_N_4_-based materials. Furthermore, g-C_3_N_4_-based photocatalysts hold significant potential for bifunctional catalysis, considering their catalytic economy and efficiency.

6. Furthermore, it is crucial to preserve and enhance the biocompatibility and eco-friendly properties of future g-C_3_N_4_-based nanomaterials.

7. To meet the industrial aim of photocatalytic hydrogen production, the solar to hydrogen (STH) efficiency must be at least 10%. Currently, the maximum efficiency attained in laboratory research is 9.2%, while the STH efficiency for g-C_3_N_4_ is less than 3%, indicating that much more work remains to be done. The most significant job for the g-C_3_N_4_ photocatalyst is to construct more efficient electron transport systems.

8. Gaining a comprehensive understanding of the underlying mechanisms driving photocatalytic H_2_O_2_ production is essential. Researchers should direct their efforts towards meticulously analyzing the various factors influencing this process, such as the adsorption dynamics of O_2_, the impact of the catalyst's surface properties on the adsorption and activation of O_2_, the intermediate stages involved in H_2_O_2_ generation, and the role of active species in modulating H_2_O_2_ production.

Despite the challenges mentioned, with continued efforts, g-C_3_N_4_-based materials still can hold great potential and limitless opportunities for large-scale environmental applications.

## Data availability

The data analyzed in this review article are from previously published studies. The specific datasets and sources are cited throughout the manuscript and listed in the reference section. Readers can access the underlying data from the original published sources as cited. The authors confirm that they did not have any special access privileges to these datasets. The data analyzed in this review article are from previously published studies. The specific datasets and sources are cited throughout the manuscript and listed in the reference section. Readers can access the underlying data from the original published sources as cited. The authors confirm that they did not have any special access privileges to these datasets.

## Conflicts of interest

There are no conflicts to declare.
